# Modified Starch Flooding in Carbonates: Impact on Oil Recovery and Crude Oil Composition

**DOI:** 10.1021/acsomega.6c04058

**Published:** 2026-07-27

**Authors:** Lorraine L. G. C. de Araujo, Luciana G. P. Sodré, Danielle Mitze Muller Franco, Leonardo dos S. Cescon, Boniek Gontijo, Georgiana F. Da Cruz

**Affiliations:** † Institute of Chemistry, Universidade Federal do Rio de Janeiro (UFRJ), Cidade Universitária, Rio de Janeiro, RJ 21941-909, Brazil; ‡ Laboratory of Petroleum Engineering and Exploration, Universidade Estadual do Norte Fluminense Darcy Ribeiro (UENF), Imboassica, Macaé, RJ 27910-970, Brazil; § Institute of Chemistry, Universidade Federal de Goiás (UFG), Goiânia, GO 74690-900, Brazil

## Abstract

Enhanced oil recovery (EOR) in carbonate reservoirs has been a persistent challenge due to their complex wettability and heterogeneity. In a previous study, the introduction of quaternary ammonium groups was shown to increase the amphiphilic character and solubility of the starch derivatives, promoting wettability alteration from oil-wet to mixed-wet conditions at concentrations of 1 mg/mL on the limestone outcrop surface. Motivated by these findings, three 2-hydroxy-3-(trimethylammonium)­propyl starch derivatives (HTPS), previously synthesized from corn starch with different cationic degrees of substitution (DS) and molar masses (MM), were evaluated as potential enhanced oil recovery (EOR) agents. Core-flood experiments on carbonate plugs revealed incremental oil recoveries of 6–11% over conventional waterflooding, with higher DS and MM enhancing oil displacement efficiency through improved carbonate wettability alteration and sweep efficiency. To provide molecular-level evidence for the oil recovery mechanisms, the original crude oil and recovered oils from core-flood tests were analyzed by ultrahigh-resolution electrospray ionization Fourier transform ion cyclotron resonance mass spectrometry (ESI(±) FT-ICR MS). The compositional analysis demonstrated significant changes in acidic and basic polar compounds, particularly within nitrogen- and oxygen-containing classes, suggesting selective adsorption and fractionation processes at the rock–fluid interface. These findings highlight ESI(±) FT-ICR MS as a key analytical tool linking macroscopic recovery performance with molecular-level fractionation in the recovered oil, thus validating the mechanisms behind the enhanced oil recovery observed in carbonate cores.

## Graphical abstract

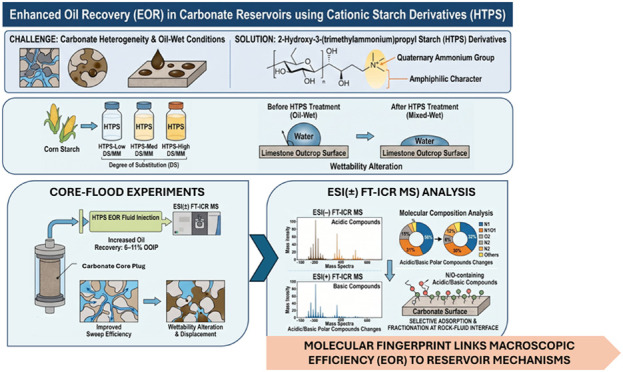

## Introduction

Although clastic reservoirs have been extensively studied in enhanced oil recovery (EOR), more than half of the world’s conventional petroleum is found in carbonate reservoirs.^1,2^ Oil recovery in carbonates is generally lower than in clastic reservoirs due to factors such as porosity and permeability being strongly dependent on diagenetic processes, rock heterogeneity, and a wide range of grain sizes, which result in a more complex fluid flow.^3^ Additionally, carbonate reservoirs are often mixed-wet or oil-wet, causing oil to adhere strongly to the rock surface and reducing mobilization by nonwetting phase injections, such as waterflooding.^4^

Xu and collaborators^4^ provide a general outlook of EOR in carbonates and highlight the importance of studying new injection media and focusing on optimizing existing technologies. One current technology is the chemical EOR method (CEOR), based on one or more chemical additive injections. These chemical additives provide the trapped oil displacement through fluids or rock physical–chemical changes in the reservoir.^5–7^ However, CEOR faces challenges including high cost, limited availability, and potential toxicity of additives.^8,9^ To overcome these limitations, future research has focused on developing additives derived from biodegradable materials, waste, or byproducts.^10^ Naturally occurring polymers, such as starch, are widely available, low-cost, and eco-friendly, making them attractive candidates for EOR applications.^11^

Starch, a carbohydrate composed of amylose (linear polysaccharide, ∼10^5^ g mol^–1^) and amylopectin (branched polysaccharide, ∼10^7^ g mol^–1^), can be chemically modified by introducing functional groups such as sulfonium, ammonium, amino, imino, or phosphonium, yielding derivatives suitable for petroleum applications.^12–15^ Evaluating a new additive generally requires some main phases of study before the field application, such as synthesis, characterization, determination of physicochemical properties, and pilot testing.^16^ Among these derivatives, 2-hydroxy-3-(trimethylammonium) propyl starch (HTPS) has shown potential in improving viscosity, altering rock wettability, and enhancing sweep efficiency in EOR.^14,17–19^ Laboratory studies demonstrated that HTPS synergistically increases viscosity when combined with partially hydrolyzed polyacrylamide (HPAM) in sand-pack core-flood experiments, reducing permeability and improving sweep efficiency more than HPAM alone. Furthermore, HTPS increased the hydrophilic character of clay minerals, indicating its potential to favor oil displacement through wettability modification.^18,19^

Previous studies^20,21^ synthesized three HTPS derivatives with different average molar masses (MMs) and degrees of substitution (DSs) and investigated their physicochemical properties relevant to EOR. The key findings included (1) emulsion stability increases with DS due to stronger electrostatic repulsion; (2) reduced MM enhances interfacial tension reduction, regardless of the DS; (3) the optimum condition for the HTPS addition increased the solution viscosity and altered the carbonate wettability obtained with the aqueous solution of HTPS 2, suggesting a directly proportional correlation of the DS and MM with the rheological and wetting properties; and (4) increased ionic strength decreased ζ-potential, IFT, viscosity, and wettability modification, while improving emulsion stability. In summary, these results highlight HTPS derivatives as promising stabilization attributes to act as shale inhibitors for water-based drilling fluids and additives for EOR applications, especially for core-flood experiments assessing individual HTPS injections in carbonate reservoirs that are still missing.

In addition to quantifying the amount of oil recovered relative to the original oil in place (OOIP), understanding molecular-level changes in the recovered oil is critical, particularly for the polar fraction containing nitrogen, sulfur, and oxygen (NSO) compounds. Although NSO-heteroatom classes typically represent <15 wt % of crude oil, they significantly influence oil properties, such as oil–water interfacial tension, producibility, refining costs, and overall economic value.^22–24^ The effect of water washing on reservoir oils has been widely investigated,^25–28^ particularly in the context of secondary and tertiary recovery operations. Waterflooding can alter oil composition by dissolving and removal of water-soluble compounds and lighter hydrocarbon fractions, thereby modifying the residual oil. However, despite extensive studies, the impact of waterflooding on the NSO-heteroatom classes remains poorly understood, especially in carbonate reservoirs. Recent studies have also highlighted that other injection fluids used in enhanced oil recovery (EOR), such as smart water, low-salinity brines, or CEOR solutions, can similarly alter the distribution and adsorption behavior of polar compounds at the rock surface, influencing wettability and recovery efficiency.^29,30^ Similarly, the effect of the biosurfactant on the oil polar fraction has been previously investigated by our research group.^31^

High-resolution mass spectrometry, particularly Fourier transform ion cyclotron resonance mass spectrometry coupled with electrospray ionization (ESI(±) FT-ICR MS), has emerged as a powerful tool for characterizing petroleum at the molecular level. This technique enables detailed analysis of heteroatom-containing polar compounds, resolving thousands of species simultaneously and providing information on heteroatom class (NSO), double-bond equivalent (DBE), and carbon number (CN). The precision (<1 ppm) and resolution (*m*/Δ*m*_50% > 350,000) of ESI(±) FT-ICR MS make it particularly suitable for studying molecular-level effects of EOR additives on oil composition.^32–36^ Therefore, depending on their heteroatom class (NnOoSs), it can separate polar oil components and by amount of unsaturation (double-bond equivalent, DBE) and carbon number (CN).^24^

The purpose of this study is 2-fold. First, core-flood experiments were performed to evaluate the effectiveness of HTPS derivatives in enhancing oil recovery from carbonate cores compared to conventional waterflooding, and the correlation among the HTPS degree of substitution (DS) and the average molar mass (MM) over the oil displacement results. Second, the fluid–rock interaction effects on oil adsorption within the carbonate and were investigated and the potential impacts of waterflooding and modified cationic starch injection on the oil polar fraction were assessed using high-resolution mass spectrometry coupled with an electrospray ionization source (ESI (±) FT-ICR MS), enabling molecular-level characterization of petroleum heteroatom classes.

## Material and Methods

### Materials

Three samples of 2-hydroxy-3-(trimethylammonium) propylamide (HTPS) with different degrees of substitution (DS) and average molar masses (MM) were previously synthesized and characterized^20,21^ and analyzed in this study as received. In the experiments, the HTPS 1, 2, and 3 solutions had a fixed concentration of 1 wt % in ultrapure water; Table 1 summarizes the HTPS properties that were used to discuss the findings of this study. Water was purified using a Milli-Q system with a resistivity of 18.2 MΩ cm (Millipore, USA). All other reagents were used without further purification. A seawater salinity (SWS) brine was prepared in ultrapure water with NaCl concentrations of 30,000 ppm with a density and a viscosity of 1030 kg/m^3^ and 9.64 × 10^–4^ Pa·s at 298 K, respectively, was used in this study. Sodium chloride (NaCl; 99.5% pure), cetyltrimethylammonium bromide (CTAB; ≥99%), *n*-heptane (99.5%), formic acid (≥98%), l-arginine­(≥98%), ammonium hydroxide (99.9%), toluene (99.8%), cyclohexane (99.9%), *n*-hexane (≥99%), dichloromethane (99.9%), trifluoroacetate (≥98%), and methanol (99.8%) were obtained from Sigma-Aldrich. The 20 cm-long and 3.8 cm-diameter Indiana limestone sample from the Bedford formation utilized in this study was supplied by Kocurek Industries Inc. (EUA). This rock sample was divided into three plugs, approximately 5.5 cm-long, used in the injection tests and designated as I3, I4, and I5, and one plug with approximately 3.0 cm thickness, used for the rock compositional analysis: elemental and mineralogical characterization and their dimensions and weights are presented in Table 5. The oil phase used in this study was a mixture (50:50 wt %) of a degassed crude oil sample from a Brazilian carbonate oil reservoir from Santos Basin; presalt was supplied by Petrobras, Brazil, with cyclohexane (99.9%) purchased from Sigma-Aldrich as a surrogate to light crude oil. The SARA analysis performed on the crude oil sample shows 34.5% saturates, 23.2% aromatics, 16.2% resins, and 8.5% asphaltenes.^21^ More details about the oil characterization are summarized in Table 2.

**Table 1:** HTPS Properties^a^

HTPS sample	MM (10^6^ g·mol^–1^)	1H-NMR DS	A2 (10^–4^ mL·mol·g^–2^)
1	1.03 ± 0.17	0.31	2.68 ± 0.23
2	1.14 ± 0.24	0.61	2.81 ± 0.16
3	0.837 ± 0.14	0.92	1.40 ± 0.18

a Characterization data reprinted in part with permission from de Araujo, L. L. G. C.; et al. Carbohydr. Polym. 2024, 323, 121388. Copyright 2024 Elsevier.

**Table 2:** Crude Oil Properties^a^

TAN (mg KOH/g)	TBN (mg KOH/g)	viscosity at 333 K (10^–4^ Pa·s)	°API
0.66	6.33	1888	23.6

SARA composition wt % (yield 82.4%)			
saturates	aromatics	resins	asphaltene
34.5	23.2	16.2	8.5

a Adapted with permission from de Araujo, L. L. G. C.; et al. Carbohydr. Polym. 2024, 323, 121388. Copyright 2024 Elsevier.

## Methods

### Indiana Limestone Core Mineralogical and Elemental Analysis

The small plug was crushed using an AMP1-M mill (AMEF, Germany) for 30 s, and then, the material passing through a 270-mesh sieve was selected for the mineralogical analysis. Then, X-ray fluorescence (XRF) analysis was performed using the S2 Ranger XRF equipment (Bruker Company, USA), operating with a 30 kV rhodium X-ray source, and the X-ray diffraction (XRD) analysis was provided by the supplier, Kocurek Industries Inc. (USA).

### Core Plug Petrophysical Characterization

After the cutting procedure, the three rock samples were dried for approximately 24 h at 333 K until reaching a constant weight. Gas porosity, pore volume, and grain density of the samples used in the oil displacement experiments were then determined using an UltraPore 300 porosimeter (Corelab Laboratories, Netherlands) and calculated with WinPore software (Corelab Laboratories, Netherlands). Subsequently, gas permeability measurements were performed with an MP-401 permeameter (Temco Inc., USA) under a confining pressure of 1000 psi and a nitrogen source pressure of 20 psi.

The rock samples were then saturated with SWS brine inside a desiccator containing silica gel and connected to a Vac V-500 vacuum pump (BUCHI, Switzerland), maintaining a pressure of 2 mbar. The samples were first semi-immersed for 6 h, after which the brine volume was increased until the rocks were fully submerged for an additional 6 h of soaking. At the end of this procedure, the saturated bulk weights were determined gravimetrically, and the liquid pore volume (Vp) and porosity (Φ) were obtained using the method described by Tiab and Donaldson.^37^

### Core-Flood Apparatus and Method

A schematic of the setup used for the displacement tests is shown in Figure 1. The system was assembled using 316 stainless-steel lines and connectors (Swagelok, USA), along with a hydrostatic horizontal core holder (Labconte, Brazil); three transfer bottles (Labconte, Brazil) one for each injected fluid: oil phase, brine, and a 1 wt % HTPS aqueous solution; a PU-2087 microflow pump (Jasco, USA) operated at constant flow, with a maximum flow rate of 50 mL min^–1^; a differential pressure transducer (Yokogawa, Japan) with a measurement range of 0–1000 psi; a 700HTP-2 manual hydraulic pump (Fluke, USA) to apply confining pressure to the system; a manometer for monitoring the confining pressure of the core holder; and several 10 mL glass beakers for collecting effluent volumes.

**Figure 1:**
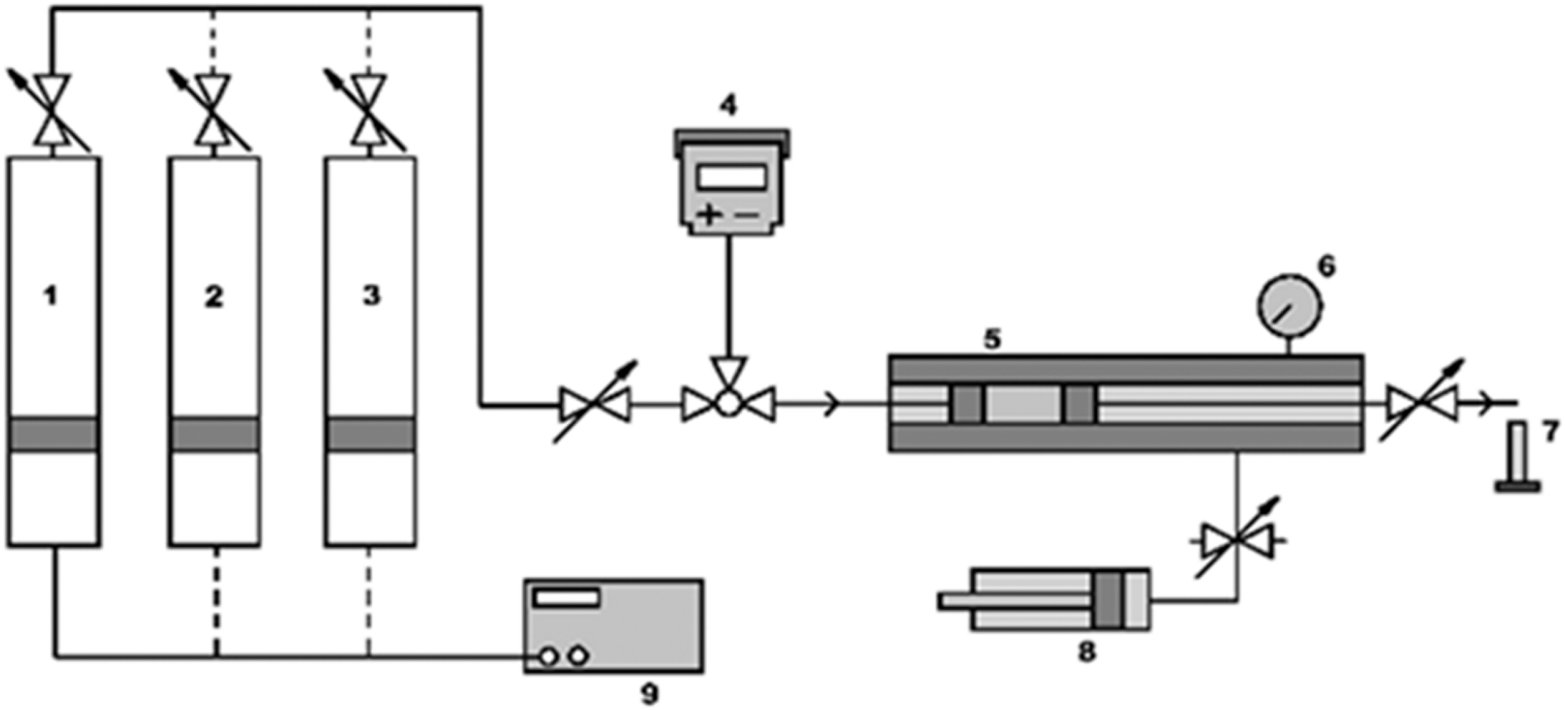
Scheme of the fluid injection system used in the core-flood experiments. (1, 2, and 3) Transfer Bottles; (4) differential pressure transducer with pressure measurement range 0 to 1000 psi; (5) hydrostatic type core holder; (6) confined pressure manometer; (7) tubes to collect the effluent; (8) back pressure valve; and (9) peristaltic pump.

According to the literature, core plugs of 1.5″ (3.81 cm) in diameter are preferred over 1″ plugs, as larger samples provide higher pore volumes and reduce measurement errors in saturation.^38^ Another important consideration to increase the reliability of core-flood test results is compliant with the Welge–JBN method,^39^ which is widely used to calculate relative permeabilities and saturations from transient-state displacement experiments in consolidated porous media.

The minimum core length (*L*_m_) required to satisfy the Welge–JBN criteria depends on rock permeability and porosity, oil viscosity, interfacial tension, and the minimum number of samples. Alternatively, for a given core length, the injection velocity can be calculated to ensure compliance with the method requirements. For the conditions evaluated in this study, the calculated minimum length was approximately 7 cm. However, due to the limited availability of the core material, three plugs with lengths of approximately 5.5 cm were used. Therefore, the injection rate of 0.5 mL·min^–1^ was calculated^40^ as the one to provide the required flow velocity to satisfy the Welge–JBN method.

The core plug was previously saturated with SWS brine and then placed inside the core holder and confined with a pressure of 1000 psi. The absolute permeability was measured as a function of the flow rate and the corresponding pressure drop based on Darcy’s law.

The procedure consisted of positioning the Indian carbonate rock sample previously saturated in SWS brine inside the core holder and the fluids in their appropriate transfer bottles. The injected oil was prepared by diluting the oil sample with cyclohexane in 50:50 (v/v) ratio to reduce its viscosity; since the test was carried out at an ambient temperature of 25 °C, the viscosity of the oil after dilution was 0.0107 Pa·s.

After connecting the entire system and saturating the injection line with SWS brine, the confining pressure of the core holder was increased, with the manual pump until reaching a stable confining pressure of 1000 psi. Next, different injection steps were performed to determine permeability to SWS brine, oil saturation until water cut, conventional recoveries (injection of SWS brine until residual oil saturation), and advanced oil recovery (injection of a pore volume of 1% wt. HTPS aqueous solution and subsequent injection of SWS brine until new residual oil saturation).

All saturations were determined volumetrically and verified by a mass balance. The injection flow used in all stages, following the determination of the absolute permeability of the rock, was 0.5 mL/min.

### Determination of Absolute Permeability of the Rock

The first step of the displacement test was the determination of the absolute permeability of the rock (k) based on Darcy’s law. As one of the faces of the rock does not have back pressure (the core holder outlet is open to the atmosphere), the pressure differential becomes only the pressure read on the pressure transducer located in the flow line. Therefore, absolute permeability to water is the angular coefficient of the straight line adjusted by the least-squares method whose points were obtained when the system was subjected to four different flow rates for constant pressure differentials.

### Determination of Initial Saturations

The second stage of the test was oil injection, with the purpose of obtaining the permeability relative to oil at an irreducible water saturation (Kro@Swi). From the volume of brine recovered, it was possible to calculate, by a mass balance, the volume of oil retained in the pores of the sample, OOIP. Next, the SWS brine was injected to obtain the relative permeability to water at the residual oil saturation (krw@Sor).

### Injection of HTPS Solutions as an EOR Method

The last step of the test was the injection of the 1 wt % aqueous solution of HTPS 1, 2, or 3, with the aim of determining the additional oil recovery provided by cationic starches. The procedure included the following steps: (1) opening the HTPS solution transfer bottle line to allow it to enter the core holder; (2) injection of a volume equivalent to 1 pore volume of HTPS 1, 2, or 3 solution; (3) opening the SWS brine transfer bottle line simultaneously with closing the HTPS solution transfer bottle line valve to allow the brine solution to enter the core holder; and (4) injection of SWS brine into the plug until the new residual oil saturation is reached.

### Calculation of the Recovery Factor

The oil recovery efficiencies obtained through seawater (SWS) injection and HTPS solution injection were quantified by using the recovery factor. The oil recovery factor (*F*_R_), also termed recovery efficiency, is calculated according to eq ([Disp-formula eq1])

1 $$F_{R}=\frac{V_{R}}{\mathrm{OOIP}}$$

where *V*_R_ is the volume of recovered oil and OOIP is the original oil in place.

To quantify the recovery achieved by enhanced oil recovery (EOR) methods, two additional parameters can be used—the incremental recovery factor (*F*_RI_) and the advanced recovery factor (*F*_RR_)—defined by eqs ([Disp-formula eq2]) and ([Disp-formula eq3])­

2 $$F_{RI}=\frac{V_{RI}}{OOIP}$$

where *V*_RI_ is the volume of oil recovered by the EOR method and

3 $$F_{\mathrm{RR}}=\frac{V_{\mathrm{RI}}}{\mathrm{OOIP}-V_{R}}$$

where the term OOIP–*V*_R_ represents the residual oil volume.

Numerically, the *F*_RR_ value is higher than the *F*_RI_ value; therefore, it is important to consider the physical meaning of each efficiency value to avoid misinterpretation. When the goal is to evaluate the total oil recovery factor (*F*_RT_) (eq 4), the *F*_RI_ is used, whereas to assess only the recovery efficiency of the EOR method, the *F*_RR_ is typically employed, since it excludes the oil recovered during the primary and secondary recovery stages.

4 $$F_{\mathrm{RT}}=F_{R}+F_{\mathrm{RI}}$$

### Recovered Oil Sample Characterization by ESI (±) FT-ICR MS

After the displacement experiments in porous medium, the recovered oils and the original oil were then characterized by positive- and negative-ion ESI modes using a 7T SolariX 2xR ultramass spectrometer (Bruker Daltonics, Bremmen, Germany). Table 3 summarizes the sample codes, extraction stages, and study objectives.

**Table 3:** Summary of the Oil Samples Analyzed by ESI(±) FT-ICR MS

sample	extraction stage	study objective
OO, original oil	crude oil before any test	blank
OA, oil adsorbed	first oil recovered in the OOIP determination	regarding the oil adsorbed into carbonate
OW, oil after waterflooding	oil recovered after the waterflooding process	regarding the water washing effect
OMS, oil after HTPS 1, 2, and 3 flooding	oils recovered after the EOR process	regarding the EOR effect

In a positive [ESI (+)] mode, oil samples (1 mg) were previously dissolved in a solution of toluene:methanol (0.67 mg·mL^–1^) (0.25% formic acid was used to aid in ionization) and infused straight into the ESI source, at a flow rate of 10 μL·h^–1^. The instrument was externally calibrated with l-arginine and settings were as follows: capillary voltage of 3.9 kV; transfer capillary temperature of 220 °C; and 2.5 L·min^–1^ drying flow. 0.04 s ion accumulation time was used, with the mass spectral scan range of *m*/*z* 200–1500, a single spectrum acquired using 200 scans, mass accuracy lower than 3 ppm, and a resolving power of about 400 000.

In a negative [ESI (−)] mode, oil samples (1 mg) were previously dissolved in a solution of toluene:methanol (0.5 mg·mL^–1^) (0.5% ammonium hydroxide was used to aid in ionization) and infused straight into the ESI source, at a flow rate of 240 μL·h^–1^. The instrument was externally calibrated with sodium trifluoroacetate (NaTFA) and settings were as follows: capillary voltage of 4.5 kV; transfer capillary temperature of 200 °C; and 4.0 L·min^–1^ drying flow. 0.010 s ion accumulation time was used, with the mass spectral range of *m*/*z* 129–2000, a single spectrum acquired using 200 scans, accuracy lower than 1 ppm, and a resolving power of 770,000.

Data processing: the mass spectra were internally calibrated with a homologous series of neutral. [ESI (−)] and basic [ESI (+)] compounds of nitrogen ions (CcHhNn), using Compass Data Analysis software (Bruker Daltonics, Bremmen, Germany) and an algorithm elaborated for petroleum signal processing (Composer software, Sierra Analytics, Pasadena, CA, US) providing the DBE versus CN plots. *M*/*z* value measurements set the elemental composition of the oil samples, and the class diagrams and DBE distribution were built in Excel spreadsheets.

## Results and Discussion

### Characterization of the Carbonate Rock Sample

#### Mineralogical and Elemental Characterization

X-ray fluorescence (XRF) results showed a CaO content of 97.0% and SiO_2_ of 1.65% and the presence of metallic cations such as Al_2_O_3_ (0.872%) and K_2_O (0.234%), which may originate from clay minerals. The mineralogical composition obtained by X-ray diffraction (XRD) was provided by the supplier, Kocurek Industries Inc. (USA). The sample was composed of 98.5% calcite, 0.7% dolomite, and 0.8% quartz. Based on the 97% CaO content and the predominance of calcite, it can be concluded that the Indiana carbonate rock sample is a calcitic limestone.^41^

#### Petrophysical Characterization

The geometric characteristics and petrophysical properties obtained from nitrogen gas (N_2_) of the core plugs used in the core-flood experiments are summarized in Table 4.

**Table 4:** Geometric and Petrophysical Properties of the Core Plugs

property	I3	I4	I5
length (cm)	5.469	5.385	5.528
diameter (cm)	3.791	3.793	3.792
dry weight (g)	138.203	136.447	139.970
porosity (%)	15.61 ± 0.04	15.88 ± 0.04	15.58 ± 0.05
grain volume (mL)	52.10 ± 0.02	51.18 ± 0.02	52.71 ± 0.03
grain density (g/mL)	2.65 ± 0.00	2.66 ± 0.00	2.65 ± 0.00
pore volume (mL)	9.64 ± 0.02	9.66 ± 0.02	9.73 ± 0.03

The average N_2_ porosity and pore volume values for plugs I3, I4, and I5 were, respectively, 15.61%, 15.88%, and 15.58% and 9.64 mL, 9.66 mL, and 9.73 mL with standard deviations of 0.13% and 0.04 mL. This sample homogeneity was essential to minimizing uncertainty in the oil displacement tests.

Most hydrocarbon reservoirs are composed of sedimentary rocks, in which porosity typically ranges from 10–40% in sandstones and from 5–25% in carbonates.^42^ Thus, the porosity of the studied plugs can be classified as good.^43^

Subsequently, gas flow (N_2_) measurements were performed to determine the pressure differences across the plugs. From the linear regression of these data, gas permeability values were obtained as the slopes of each fitted line (Figure 2).

**Figure 2:**
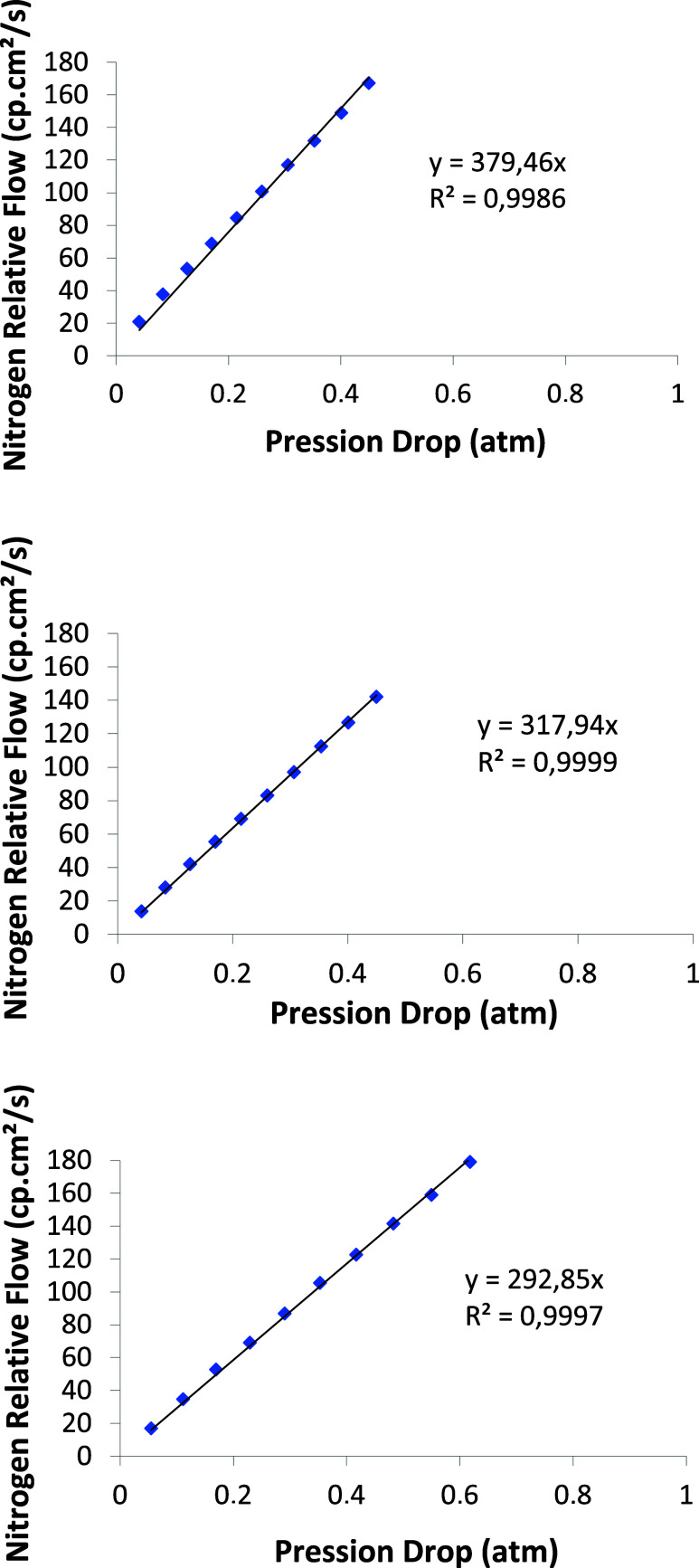
Linear regression of N_2_ flow data for samples I3, I4, and I5.

The gas permeability values for samples I3, I4, and I5 were 317.94 mD, 292.85 mD, and 379.46 mD, respectively. It is important to note that porosity and permeability are not directly correlated as pore connectivity plays a major role in governing fluid transport within the rock matrix. This behavior can be observed in sample I5, which, despite presenting slightly lower porosity values, exhibited the highest gas permeability among the tested plugs.

After completing the N_2_ petrophysical characterization, the plugs were successfully saturated with SWS brine, and porosity and pore volume were measured by the gravimetric method, and these results are presented in Table 5. These parameters were used to define the experimental conditions and ensure consistency among the core-flood tests.

**Table 5:** Petrophysical Properties Using SWS Brine for Plugs I3, I4, and I5

property	I3	I4	I5
porosity (%)	16.10	16.00	15.90
pore volume (mL)	9.96	9.72	9.95

The mean standard deviations for porosity and pore volume were 0.07% and 0.10 mL, respectively, indicating a low variability among the three plugs measured with SWS brine. Differences between the gas and liquid (brine) measurement results can be attributed to the larger uncertainty associated with the gravimetric method used to calculate liquid-phase properties.

### Core-Flood Experiment Results

This section presents the results obtained from the core-flood tests conducted to evaluate the potential of HTPS structures to enhance oil recovery in carbonate rocks. Prior to these experiments, several preliminary tests were performed to verify the proper assembly of the equipment and the reliability of the methodology. For the sake of clarity and consistency, only the results obtained using aqueous HTPS solutions are reported in this study.

#### Determination of Absolute Permeability

In the first stage of the flow tests, the absolute permeability of each rock sample was determined based on Darcy’s law. Four different flow rates (2, 4, 6, and 8 mL·min^–1^) were applied, and the corresponding pressure differentials were recorded. It should be noted that these flow rates are higher than the critical injection velocity proposed by Gruesbeck and Collins,^44^ which could lead to fines migration during this stage due to the presence of clay minerals in the rock samples. Performing this initial step under such conditions helps prevent potential fines migration in the subsequent test stages, which could otherwise increase uncertainties in the recovery factor results. Figures 3, 4, and 5 show the linear regressions for samples I3, I4, and I5, respectively. The coefficients of determination (*R* ^2^) close to 1 indicate that there was no reduction in permeability or disproportionate pressure increase with varying flow rate.

**Figure 3:**
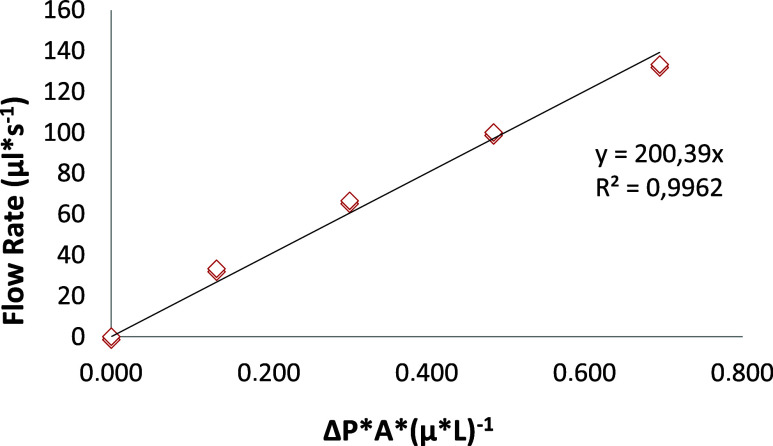
Linear regression of injection data for sample I3.

**Figure 4:**
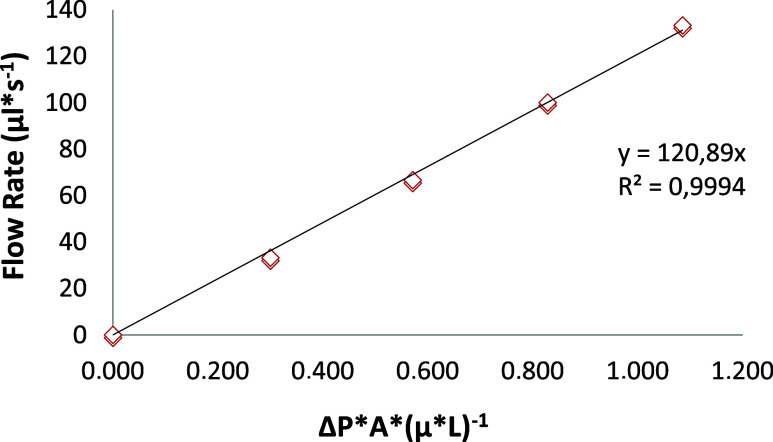
Linear regression of injection data for sample I4.

**Figure 5:**
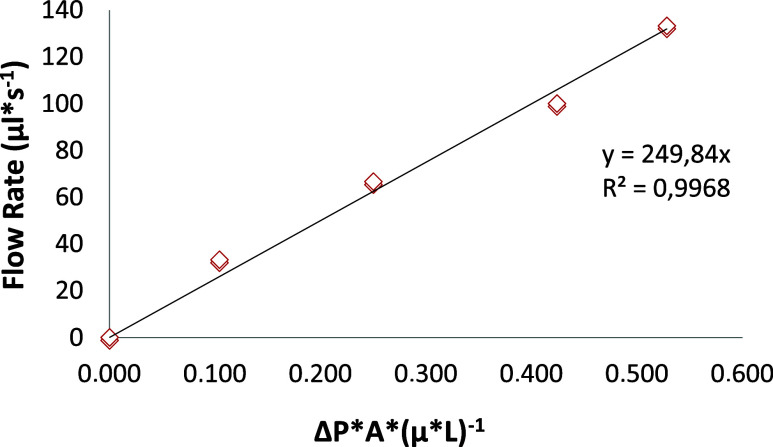
Linear regression of injection data for sample I5.

The absolute permeabilities measured through SWS brine injection for samples I3, I4, and I5 were 200.39 mD, 120.89 mD, and 249.84 mD, respectively. These results show that liquid-phase permeabilities were lower than those measured with gas, a difference attributed to the Klinkenberg effect.^45^ Regardless of the method, all samples exhibited permeability values above 100 mD, classifying them as highly permeable rocks (100–1000 mD).^46,47^ Mahesar and colaborators^45^ also observed that samples with lower overall permeability exhibited a greater relative reduction when measured under liquid flow. In addition to the Klinkenberg effect, the mobilization and dissolution of particles and other pore-blocking phenomena can further reduce liquid-phase permeability.

#### Determination of Irreducible Water Saturation (Sw_i_)

After determining the absolute permeability through SWS brine injection, the mixture of crude oil and cyclohexane was injected to displace the aqueous phase. Figures 6, 7, and 8 show the cumulative production of the aqueous phase and the pressure gradient during oil-phase injection for carbonate samples I3, I4, and I5.

**Figure 6:**
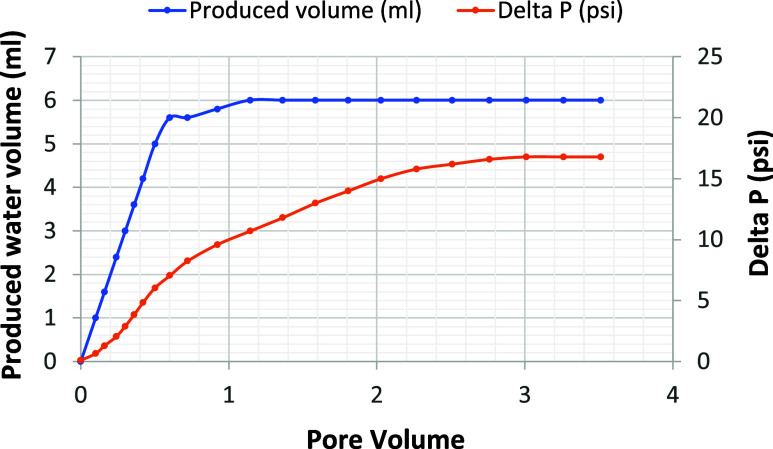
Oil-phase injection results for sample I3.

**Figure 7:**
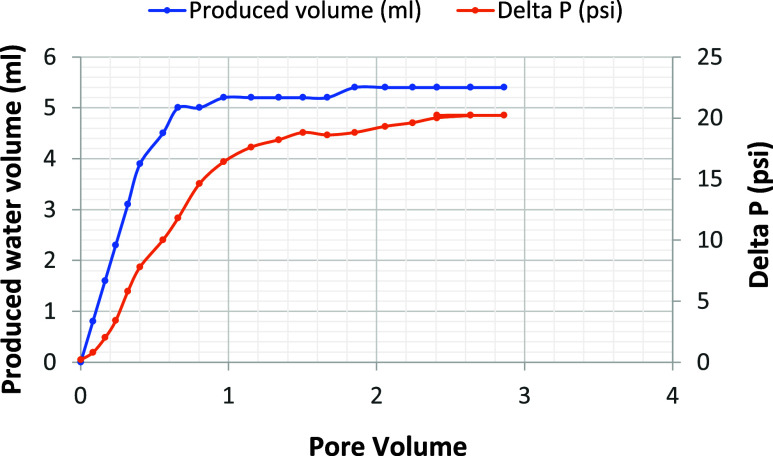
Oil-phase injection results for sample I4.

**Figure 8:**
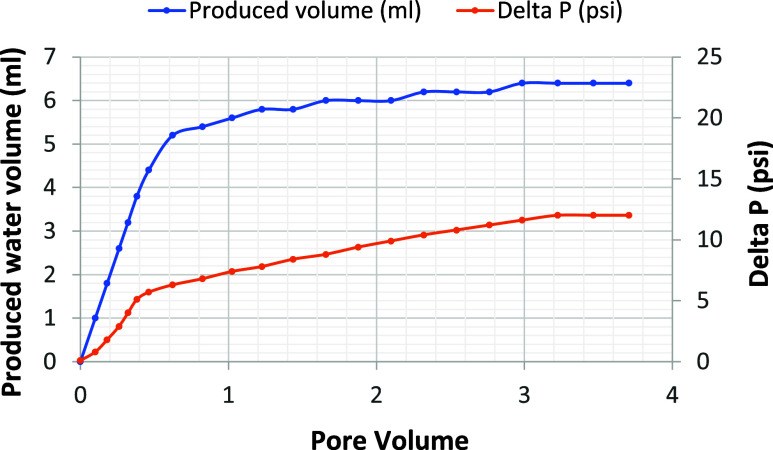
Oil-phase injection results for sample I5.

The breakthrough profiles of the aqueous phase displaced by oil injection were similar for all three samples with differences only in the final produced volumes. The calculated irreducible water saturations (Sw_i_) were 39.8%, 44.5%, and 35.7% for samples I3, I4, and I5, respectively.

However, the pressure differential (Δ*P*) profiles displayed different slopes among the samples, with final Δ*P* values of 16.8, 20.2, and 12 psi, respectively. These results can be attributed to differences in absolute permeability (*k*), which represents the degree of pore connectivity governing multiphase flow. The higher pressure differentials observed in sample I4 are consistent with its lower absolute permeability (120.89 mD).

#### Primary Recovery and Enhanced Recovery by SWS Brine and HTPS Injections

HTPS 1, 2, and 3 solution injections were carried out using plugs I4, I3, and I5, respectively. Figures 9, 10, and 11 show the oil recovery versus injected pore volume curves for the three experiments.

**Figure 9:**
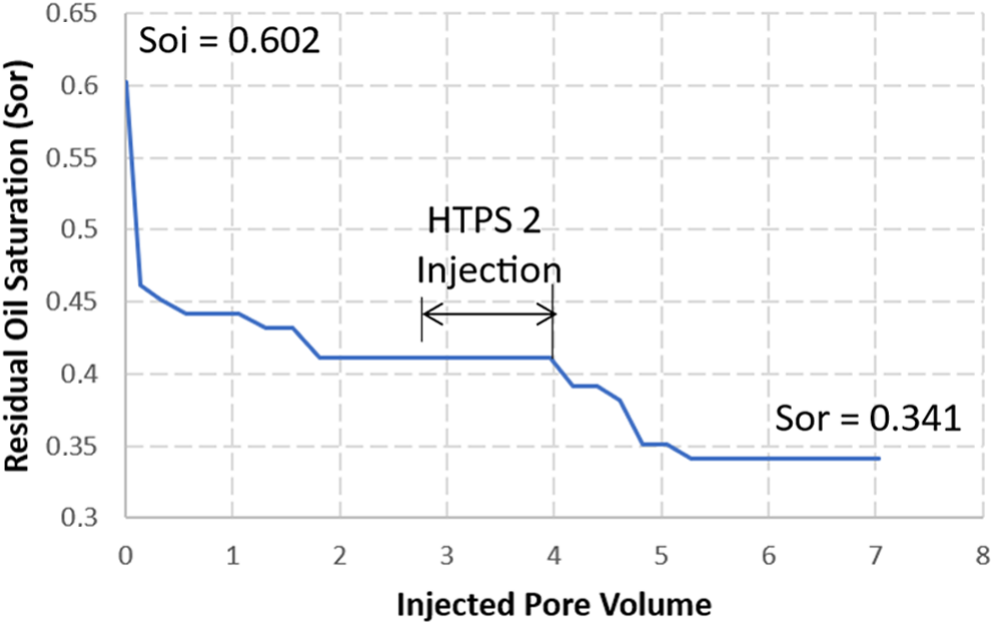
Oil recovery for sample I3 resulting from SWS brine, HTPS, and post-SWS brine injections.

**Figure 10:**
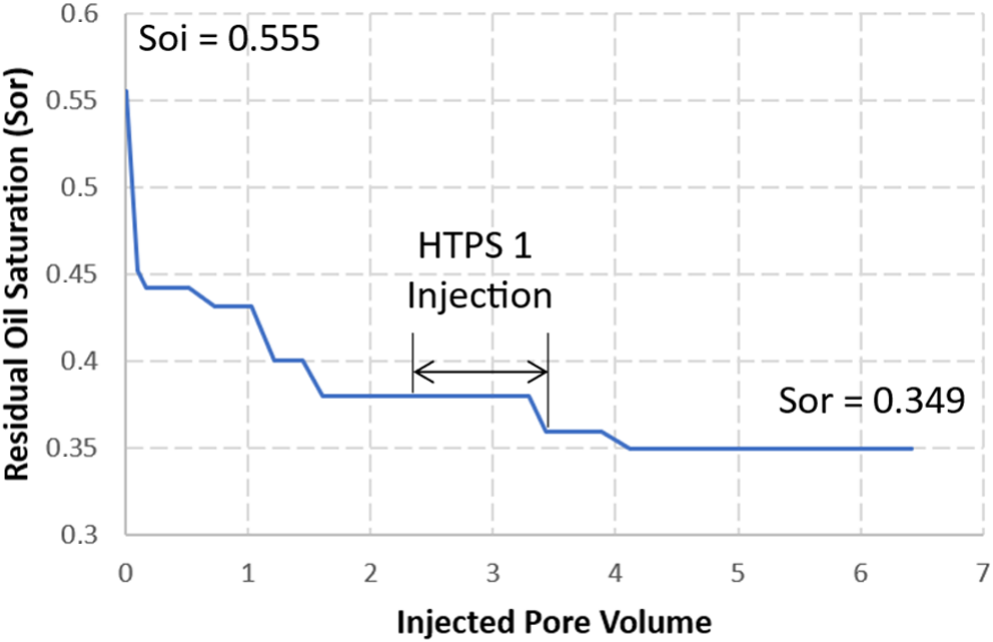
Oil recovery for sample I4 resulting from SWS brine, HTPS, and post-SWS brine injections.

**Figure 11:**
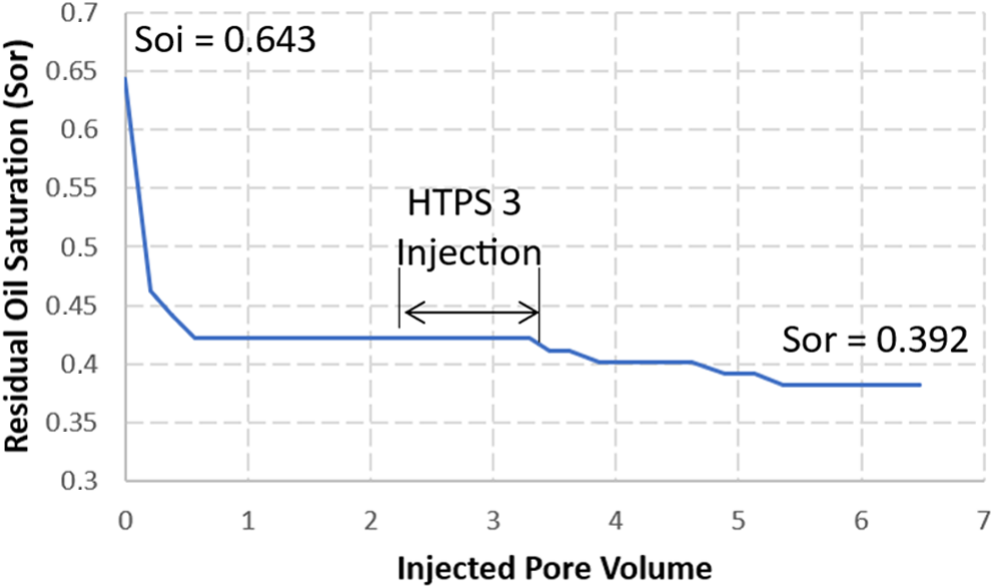
Oil recovery for sample I5 resulting from SWS brine, HTPS, and post-SWS brine injections.

The initial oil recovery factors (FRs) achieved through SWS brine injection were 31.6%, 31.5%, and 34.3% OOIP for samples I3, I4, and I5, respectively. According to the literature, average recovery factors in carbonate reservoirs are typically below 35%.^8^

In all three experiments, the aqueous-phase breakthrough occurred at approximately 0.2 pore volumes (PVs) injected. The residual oil saturations (Sor) calculated after the SWS brine injection were 41.2%, 38.1%, and 42.2%, respectively.

Figures 9–11 also show the performance of the aqueous solutions of HTPS 2, HTPS 1, and HTPS 3 (1 wt %) in the respective samples. After the primary recovery plateau (around 2 PVs), oil production resumed approximately 0.9 PV after HTPS injection began. This delay corresponds to the time required for the oil bank to form and advance, which depends on the interaction time between each HTPS solution, the oil, and the rock surface.

The injection of the HTPS 1, 2, and 3 solutions (1 PV each) resulted in incremental recovery factors (FRI) of 5.5%, 11.7%, and 6.3% OOIP, respectively, increasing the total oil recovery factor (FRT) to 37.0%, 43.3%, and 40.6% OOIP at the end of the entire injection process (Figure 12). The HTPS injections also reduced the residual oil saturation (Sor) to 34.9%, 34.1%, and 39.2%, respectively.

**Figure 12:**
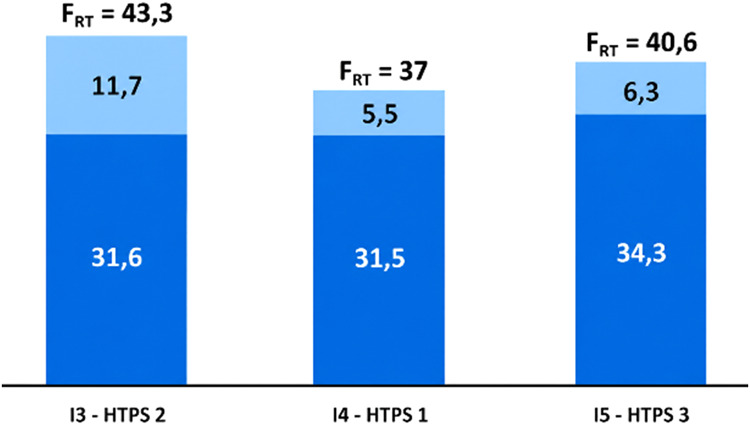
Comparison of oil recovery factors (FR, FRI, FRT).

Figure 13 shows the pressure differential (Δ*P*) profiles observed during the oil recovery processes. The Δ*P* values measured at the end of SWS brine injection (primary recovery) were 9, 11, and 6 psi, respectively, another indicator of the differences in carbonate rock permeability.

**Figure 13:**
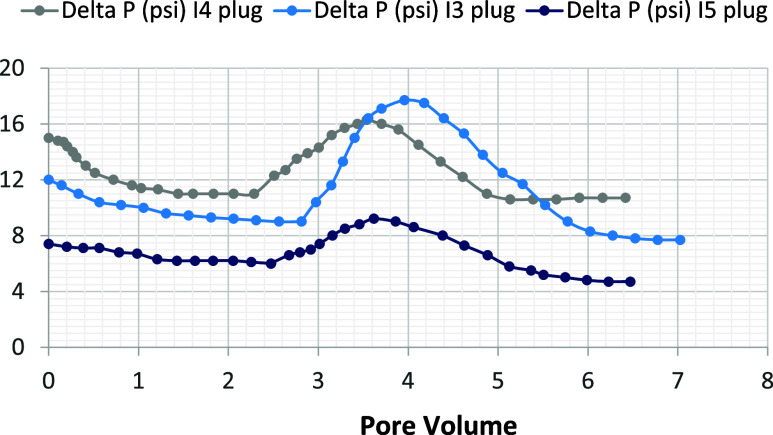
Pressure differential (ΔP) profiles during oil recovery for samples I3, I4, and I5.

As expected, Δ*P* values increased during HTPS injection and reached their maximums at 17.7, 15.7, and 9.2 psi, respectively, at the end of 1 PV of HTPS injection. Due to the higher viscosity of the HTPS 2 solution (injected in sample I3) compared to HTPS 1 and HTPS 3, the maximum Δ*P* observed for this sample exceeded that of sample I4, which had the lowest absolute permeability among the three carbonates. At the end of the HTPS and subsequent SWS injections, the measured Δ*P*s were 7.7, 10.7, and 4.7 psi, respectively, due to the reduced oil saturation in the cores.

In summary, the results of these three experiments demonstrate the effectiveness and efficiency of the HTPS derivatives studied for enhancing oil recovery. HTPS 2 and HTPS 3 outperformed HTPS 1, consistent with previous results^21^ that suggest that wettability alteration must be primarily responsible for this additional oil recovery achieved.

The results of the characterization of the oil used are presented in Table 2. According to petroleum density classification,^48^ the API gravity of 23.6 is characteristic of a medium crude oil. The TAN value of the sample (0.66 mg KOH/g) indicates an acidic crude oil (0.5 < TAN < 1.0 mg KOH/g),^49^ while the TBN value of 6.33 mg KOH/g was also relatively high. These results indicate the significant presence of ionizable acidic and basic species, such as resins and asphaltenes, which may contribute to electrostatic phenomena at interfaces influencing processes such as wettability alteration in carbonate rocks.^50,51^

In this context, another mechanism of wettability alteration in carbonates reported in the literature is the surface precipitation of asphaltenes. Resins (R) are known to play a key role in stabilizing asphaltenes (A) and preventing their precipitation, particularly when the A/R ratio is less than 0.35.^52^ The calculated A/R ratio for the sample was 0.52, indicating that this crude oil presents a tendency toward instability from a flow-assurance perspective. Therefore, the contribution of asphaltenes to the observed interfacial phenomena must be carefully evaluated.

## Petroleomic Results

### Basic Polar Compounds by ESI (+) FT-ICR MS

The oil samples were ionized by ESI (+) FT-ICR MS to investigate changes in the basic polar compounds (nitrogen-containing species), once positive ions in FT-ICR MS can detect a wide range of carbon numbers for alkyl dibenzothiophenes from C0 to C35.^53,54^

For consistency with the figure labeling and to facilitate the discussion of the influence of the different modified starches, the oil samples (OO, OA, OW, and OMS; details in Table 3) obtained from the core-flood tests will hereafter be referred to according to the respective HTPS systems. Thus, the oils from the tests conducted in plugs I3, I4, and I5 will be designated as oils from the HTPS2, HTPS1, and HTPS3 tests, respectively. This nomenclature is adopted from now on to simplify the comparison among experiments and to improve the clarity of the discussion.

It is important to emphasize that the original oil (OO) corresponds to the oil sample collected prior to the core-flood experiments and, therefore, should not exhibit any influence from the injection processes or differences among the tests. The adsorbed oil (OA) reflects the influence of oil adsorption within the porous medium, while the oil recovered with brine (OW) represents the effect of the water washing process. In contrast, the oil recovered with modified cationic starch (OMS) is used to evaluate the influence of the physicochemical properties of the injected polymers, particularly their molecular weight and degree of cationic substitution (details provided in Table 1), on the additional displacement of the trapped oil.

The compounds detected by mass spectrometry were organized and divided according to their heteroatom classes (NnOoSs), being convenient to analyze the relative proportion between them.^55,56^ As shown in Figure 14, the total species detected in the oil samples of each core-flood test are composed of N, N_2_, N_2_O, NO, NOS, and NS, considering only those detected above 1% of the total relative abundance. Classes detected below 1% were grouped and identified as others; additional heteroatom species are likely present but fall below the signal-to-noise threshold required for reliable molecular formula assignment.

**Figure 14:**
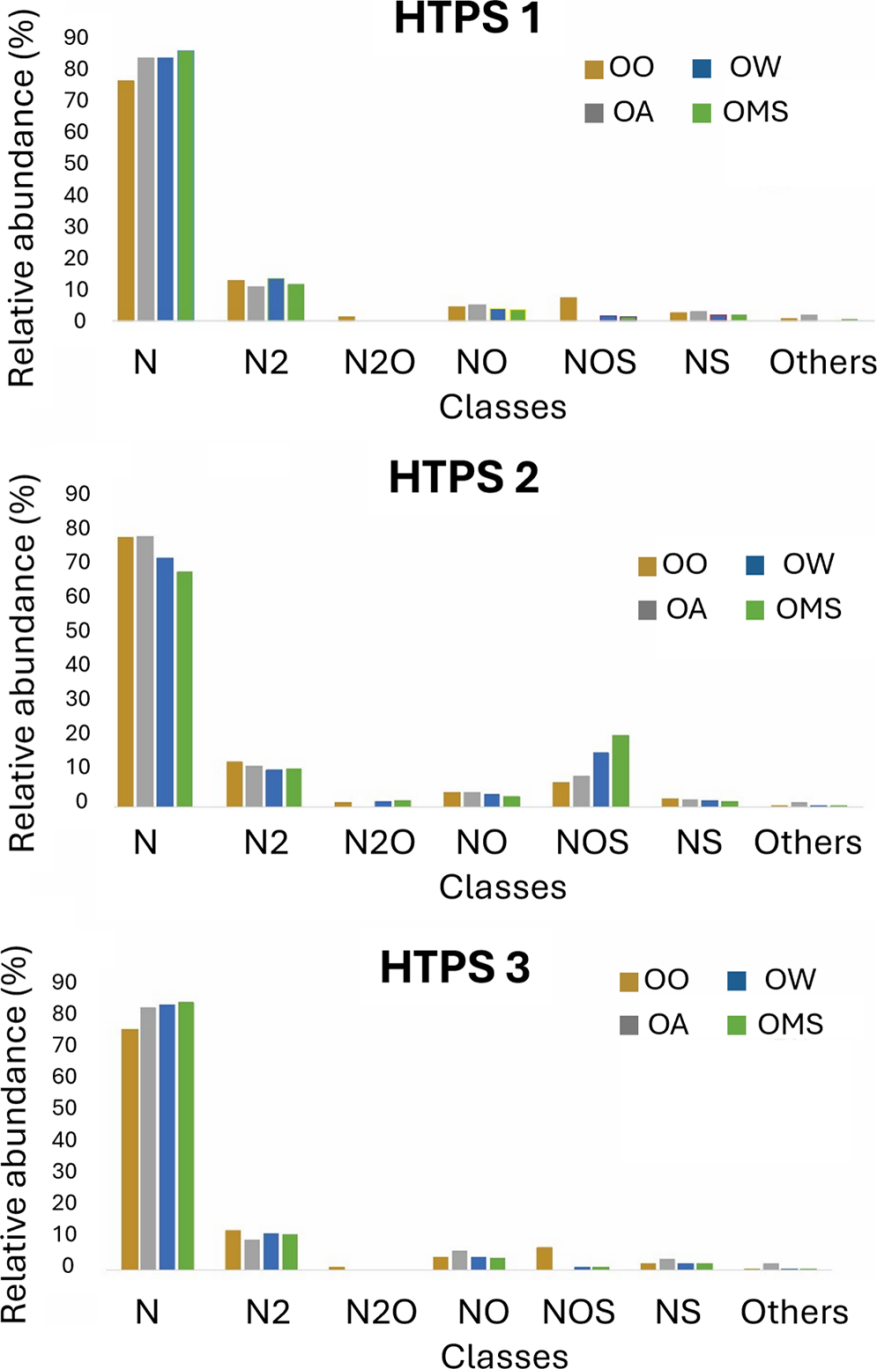
HTPS 1, HTPS 2, and HTPS 3 test representative cumulative abundances for major species detected by the positive-ion mode in FT-ICR MS.

Overall, the analyzed oils have a similar species distribution and relative abundances, particularly in the original oil (OO), which is not subjected to any core-flood influence. The N species is the predominant chemical class (63% to 82.3% of the total identified species) in all oils across the three core-flood tests.

The HTPS 1 and HTPS 3 oils exhibit similar heteroatom class distribution profiles for OA, OW, and OMS, with OMS showing slightly higher relative abundance. In contrast, the original oil (OO) indicates an increasing trend in the N class in oil samples after the core flooding tests and a decrease or even suppression of the NOS class. This behavior was particularly evident for OA, indicating a strong preferential adsorption of these compounds onto the carbonate matrix, with only a slight recovery observed during the SWS and EOR injection.

However, as shown in Figure 14, HTPS 2-adsorbed oil (OA) more closely resembled the original oil (OO). In this core-flood test, oils were subjected to a distinct geochromatographic process, and a key factor could be associated with potential variations in the mineralogical composition^57^ of the plugs (e.g., clays and silica). OW and OMS show slight enrichment in N2O class compounds and, more notably, to the NOS class, the latter being the most abundant class in this test, particularly in the oil recovered with modified cationic starch HTPS 2. These results corroborate the findings reported in the previous work,^21^ as well as the recovery factor presented in the previous section.

Additionally, further insights into individual heteroatom species (N and N2) can be obtained by examining the DBE distribution plots (Figures 15, 16, 17).

**Figure 15:**
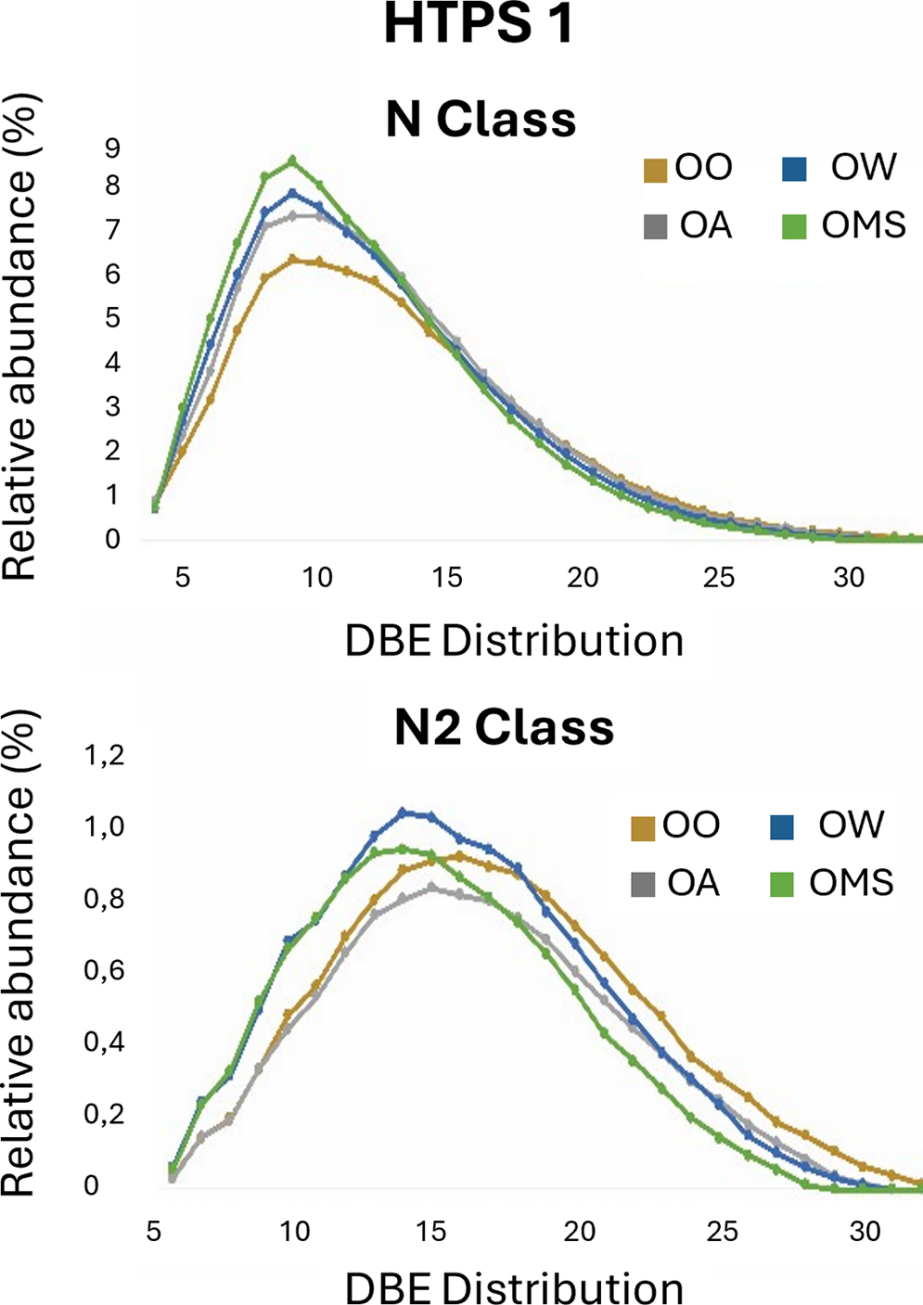
HTPS 1 DBE distributions plots of 1 N class and N2 class.

**Figure 16:**
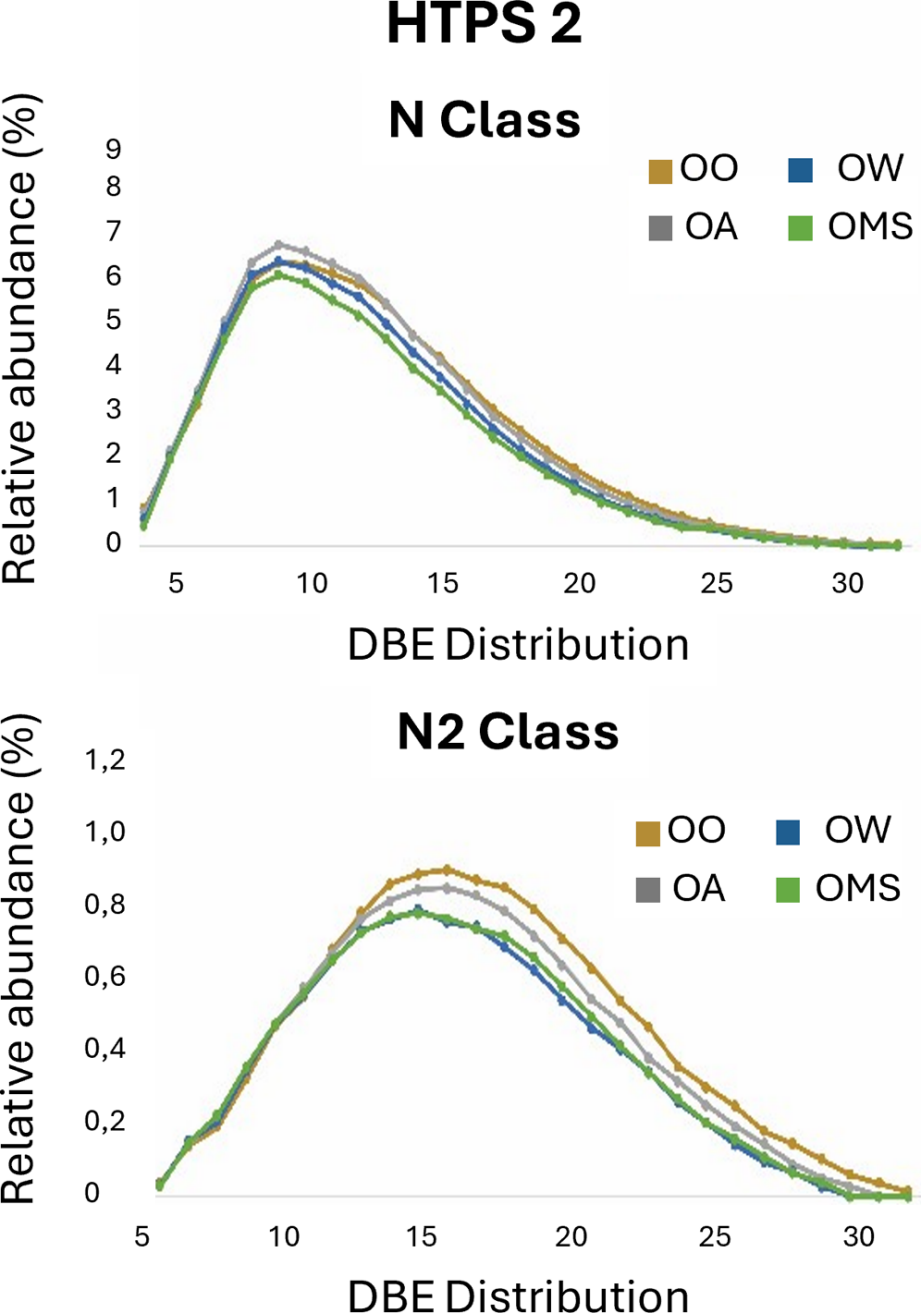
HTPS 2 DBE distributions plots of 1 N class and N2 class.

**Figure 17:**
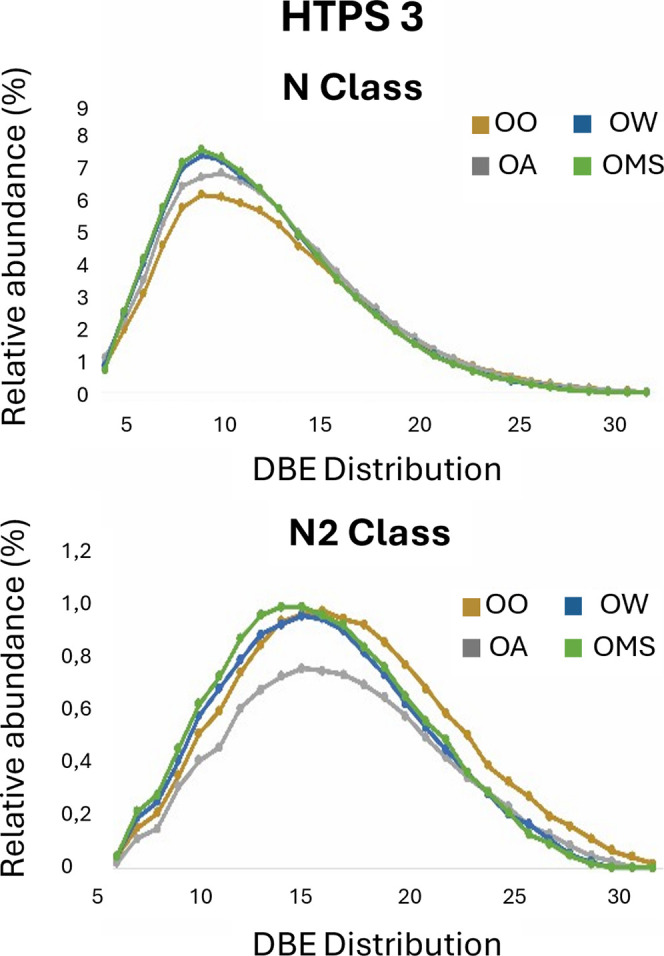
HTPS 3 DBE distributions plots of 1 N class and N2 class.

Regarding the N class, the DBE distributions for HTPS 1 and HTPS 3 (Figures 15 and 17) show an increase in the relative abundances of compounds with DBE values between 5 and 14 in the OA, OW, and OMS oils when compared to the original oil (OO), particularly for OMS in HTPS 1, in which compounds with DBE values up to 10 were the most affected by the enhanced oil recovery experiment.

On the other hand, the DBE distribution for HTPS 2 (Figure 16) shows a more pronounced decrease in the relative abundances of compounds with DBE values between 8 and 23 for OMS compared to those for OO. The OW and OA samples showed only subtle changes in their DBE distributions when compared with the original oil. In this context, it is important to note that the reductions in the relative amounts of N compounds observed in HTPS 2 for OW, and especially for OMS (Figure 14), are clearly associated with medium- to high-DBE compounds ranging from 13 to 31. This behavior indicates a tendency toward reduced aromaticity in these oils in HTPS 2 relative to that of the original oil and even to the other tests.

In addition, these graphs indicate that across all tests, DBE 9 was, overall, the most abundant species in the analyzed oils. N species with DBE values of 9, 12, and 15 determined under ESI (−) FT-ICR MS are most likely associated with carbazoles, benzocarbazoles, and dibenzocarbazoles, respectively.^53,57^ However, because of their weaker basicity, these compounds are not efficiently detected under ESI (+) conditions. Therefore, the N species with DBE values of 9, 12, and 15 detected in positive-ion mode are more likely to correspond to quinolines substituted with naphthenic side chains, benzoquinolines, and dibenzoquinolines bearing alkyl or naphthenic side chains, respectively.^53,57^

Figure 18, therefore, presents the relationship between DBE 9 and DBE 12 (selected because of their higher relative abundances compared to DBE 15), allowing a more in-depth analysis of the oils recovered with brine and with modified cationic starches (OW and OMS) in comparison with the original oil (OO) across all tests.

**Figure 18:**
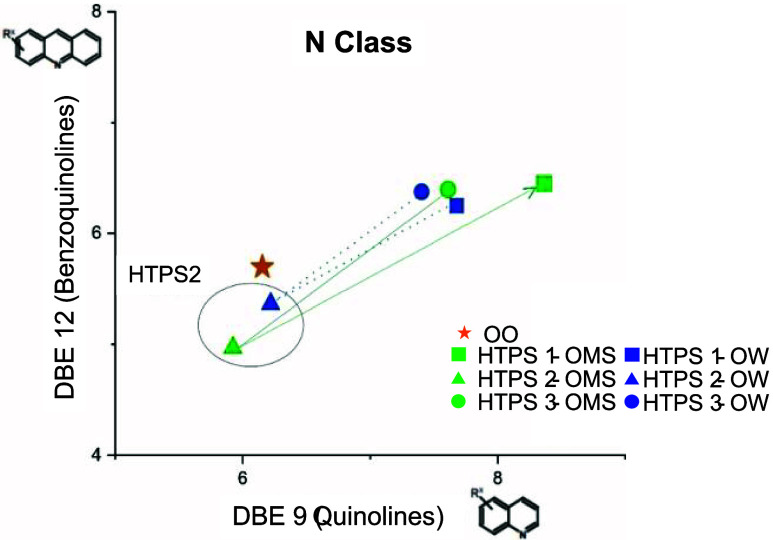
Relationship of relatively abundant DBE 9 and DBE 12 from oils recovered with brine (OW) and oils recovered with modified cationic starches (OMS) of the core-flood tests, compared to original oil (OO).

Figure 18 shows a decrease in their relative abundances for OW and OMS for the HTPS2 test, compared to the original oil (OO). In contrast, both OMS for HTPS 1 and 3 tests exhibit a more pronounced increase in the relative abundances of these DBE species.

Nitrogen compounds in petroleum affect several important refining processes;^58^ therefore, from this perspective, HTPS 2 would be the most suitable modified starch, since it shows the lowest relative abundances of these species compared to the other systems evaluated in this work.

### Acidic Polar Compounds by ESI (−) FT-ICR MS

The oil samples (OO, OA, OW, and OMS) were also assessed by ESI (−) FT-ICR MS to investigate changes in acidic polar compounds, particularly nitrogen- and oxygen-containing species. All results for each core-flood test (HTPS 1, HTPS 2, and HTPS 3) are presented in this section, as well as the relationships among them. The heteroatom class distribution (NnOoSs) of the original oil and the recovered oils from the tests is shown in Figure 19, displaying all classes with relative abundances above 1%.

**Figure 19:**
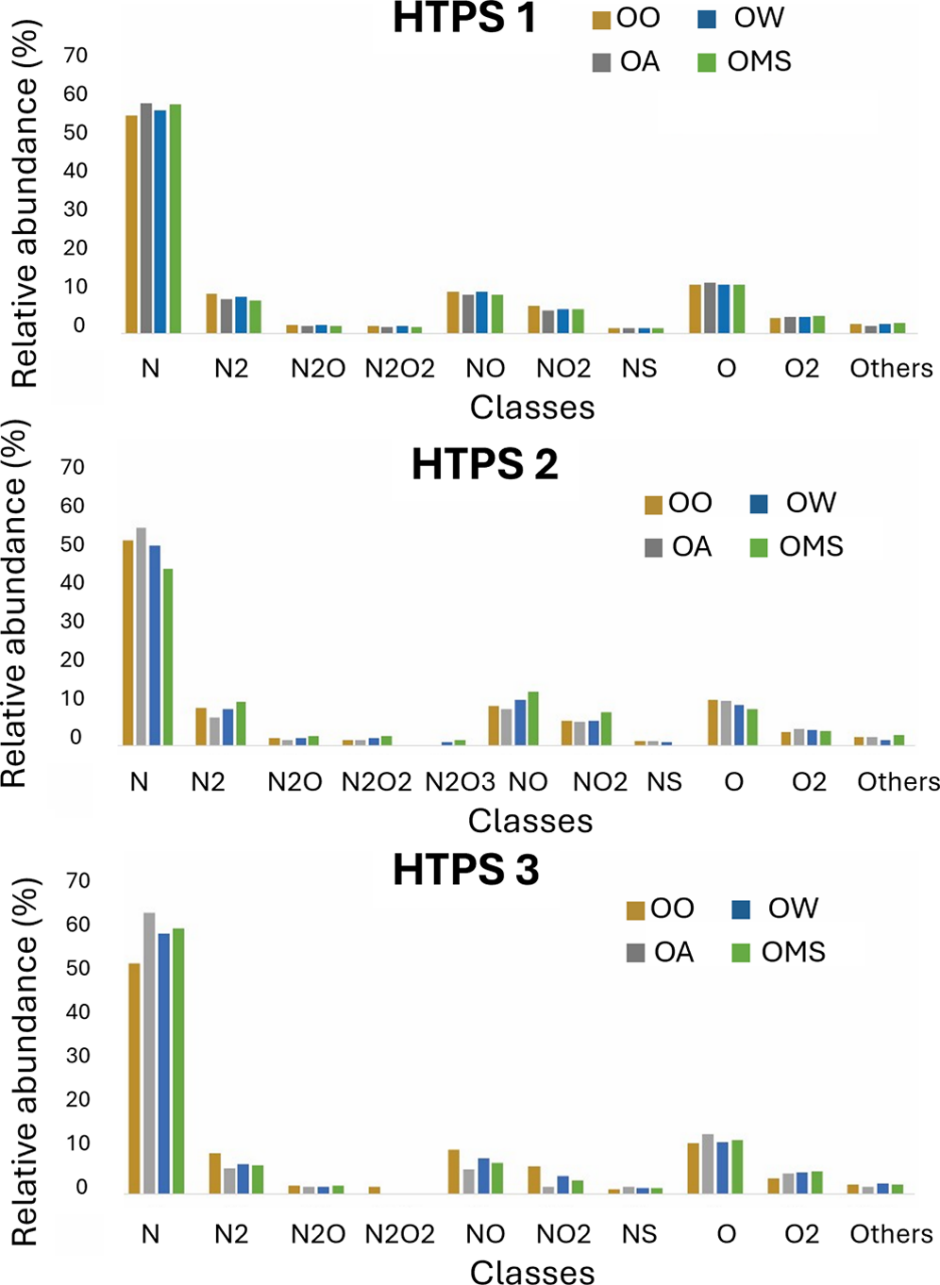
HTPS 1, HTPS 2, and HTPS 3 test representative cumulative abundances for major species detected by the negative-ion mode in FT-ICR MS.

According to Figure 19, HTPS 1 and HTPS 3 show overall similarities in their heteroatom class distribution profiles, particularly for the N, O, and O2 classes. The recovered oils (OW and OMS) generally show an increase in the relative abundances of N, O, and O2 classes compared to the original oil (OO), with OA presenting the highest relative abundances in the N and O classes and OMS showing the highest relative abundance in the O2 class. Conversely, there is a trend toward a decrease or even suppression of other classes in the recovered oils (OWs and OMSs) relative to the original oil.

The N class exhibits the highest abundance among all oils (HTPS 1, HTPS 2, and HTPS 3), and the relative ion abundances of N and O classes decreased with the ongoing HTPS 2 core-flood test (particularly in the oil recovered with modified cationic starch). In contrast, the relative ion abundances of N2, NO, and NO2 classes exhibited an increasing trend.

Thus, the recovered oils (OW and OMS) of the three tests were also assessed in a ternary diagram (Figure 20) and compared to the original oil (OO), using the relative amounts of the three major elementary classes, Nx, Oz, and NxOz, to further investigate the relation of these classes to each other, regarding the relative abundances of these compound groups.

**Figure 20:**
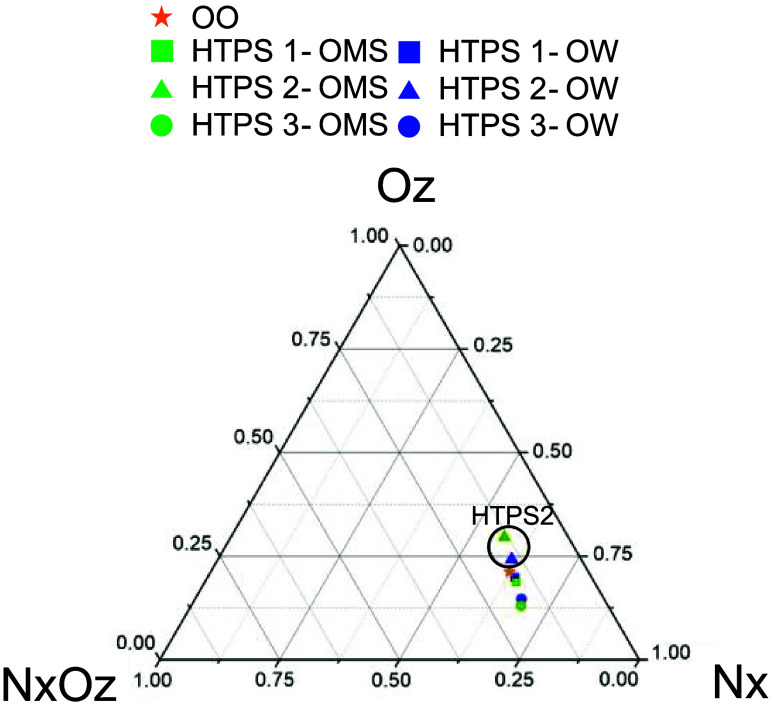
Ternary diagram for the most abundant elemental classes assigned on the negative ESI FTICR MS results: Nx, Oz, and NxOz, for the original oil (OO) and the recovered oils (OW and OMS) in all tests.

These results suggest differences in the distribution of the oil samples’ polar compounds. Despite all of them showing a tendency toward enrichment in Nx compounds, HTPS 2 once again shows itself to be more different, with the lowest concentrations of this compound group, mainly for OMS. Interestingly, in this analysis, the recovered oils of HTPS 1 were more similar to the OO. The recovered oils in HTPS 3 more enriched in Nx, mostly in OMS.

The O2 class, despite not being highly abundant in the oils analyzed in the three tests, represents an important group in studies of the formation of stable emulsions. Pereira and collaborators^60^ reported that acidic polar compounds contribute to the formation of stable emulsions when present in produced oils. They demonstrated, for example, that crude oils with API gravities ranging from 26 to 31 and low total acid number (TAN) values (0.03 to 0.57 mg of KOH/g) exhibited high O/O2 ratios and were associated with the formation of more stable emulsions. Although the crude oil studied in this work presents a slightly lower API gravity and a somewhat higher total acid number (TAN; details in Table 2) than the ranges reported in the literature,^60^ the O/O2 ratios were calculated for the oils recovered through brine injection and modified starch flooding in the three core-flooding tests, in order to evaluate the potential implications for stable emulsion formation under the conditions investigated (Table 6).

**Table 6:** O/O_2_ Ratios Calculated for Oils Recovered

test	oil sample	O/O_2_ ratio
HTPS 1	OW	3.05
HTPS 1	OMS	2.73
HTPS 2	OW	2.51
HTPS 2	OMS	2.41
HTPS 3	OW	2.42
HTPS 3	OMS	2.32

The O/O2 ratio values calculated for oils recovered in HTPS 2 and HTPS 3 are relatively similar. These results indicate that the higher relative abundances of O and O2 classes in the oils recovered in HTPS 1 (especially for OW) may contribute to stable emulsion formation; however, HTPS 3 subtly showed the lowest values (especially for OMS). These results may be interpreted as a positive indicator for reducing the risk to the former stable emulsion and, consequently, a lower impact on flow assurance compared to the oils from the HTPS 1 test.

Further insights into individual heteroatom species (N and O) can be obtained by examining the DBE distribution plots (Figures 21, 22, and 23).

**Figure 21:**
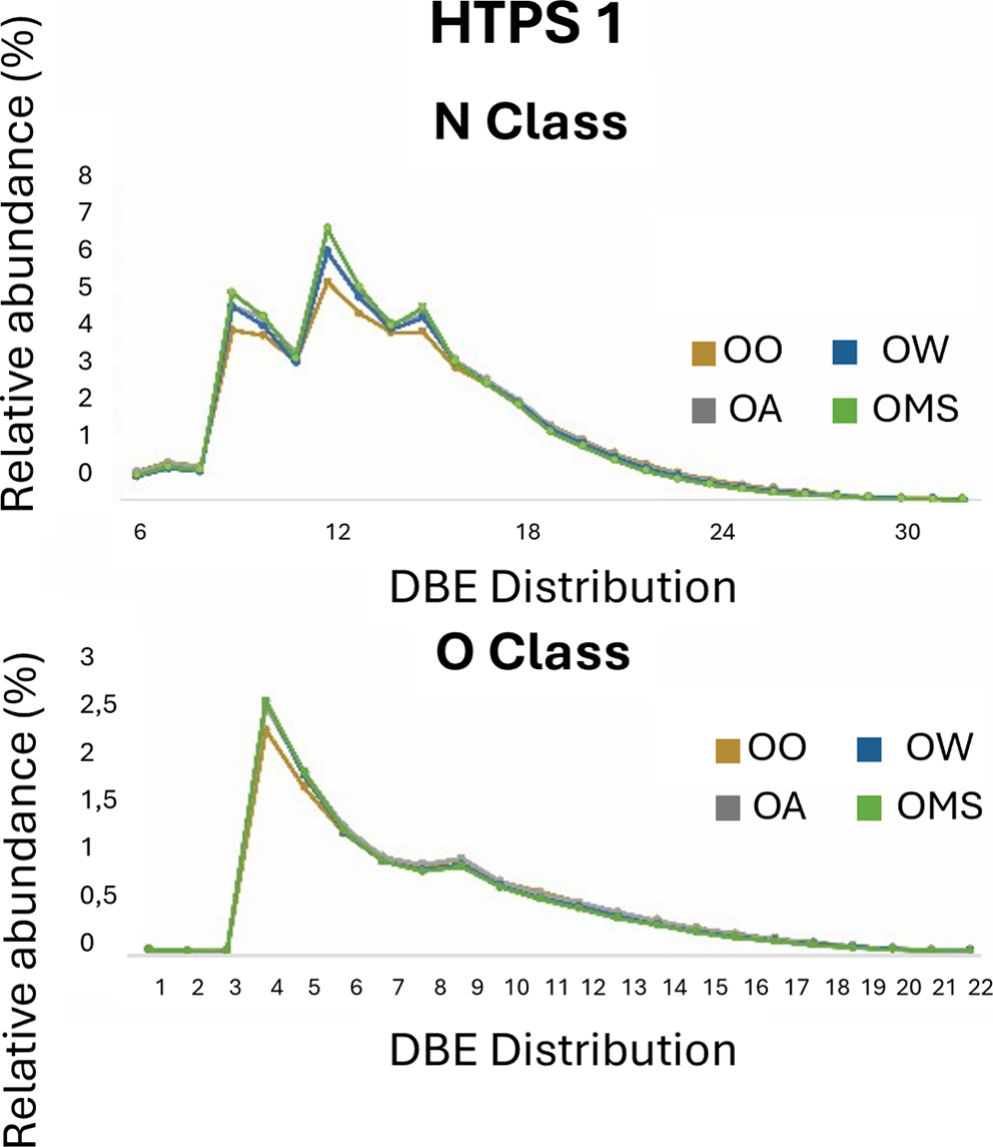
HTPS 1 DBE distribution plots of the 1 N class and O class.

**Figure 22:**
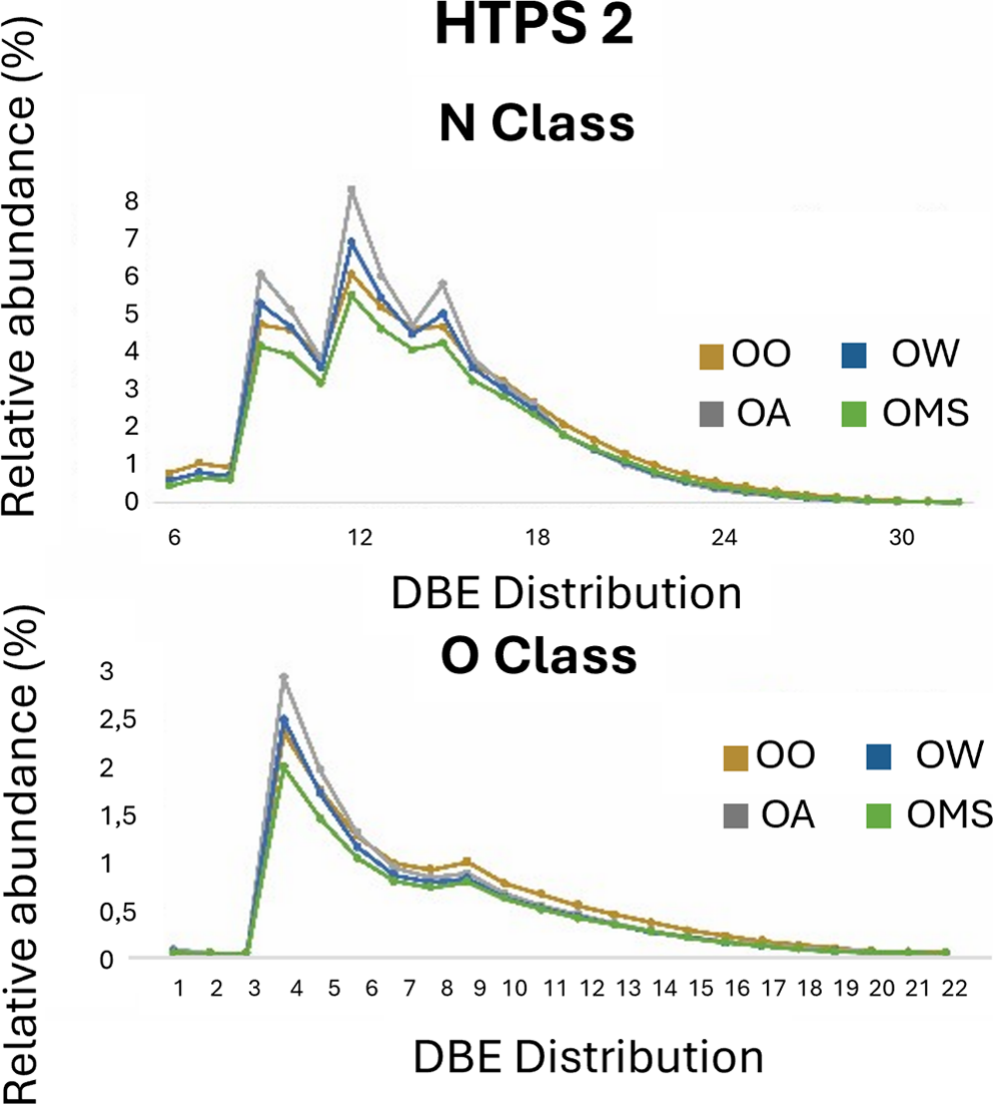
HTPS 2 DBE distribution plots of the 1 N class and O class.

**Figure 23:**
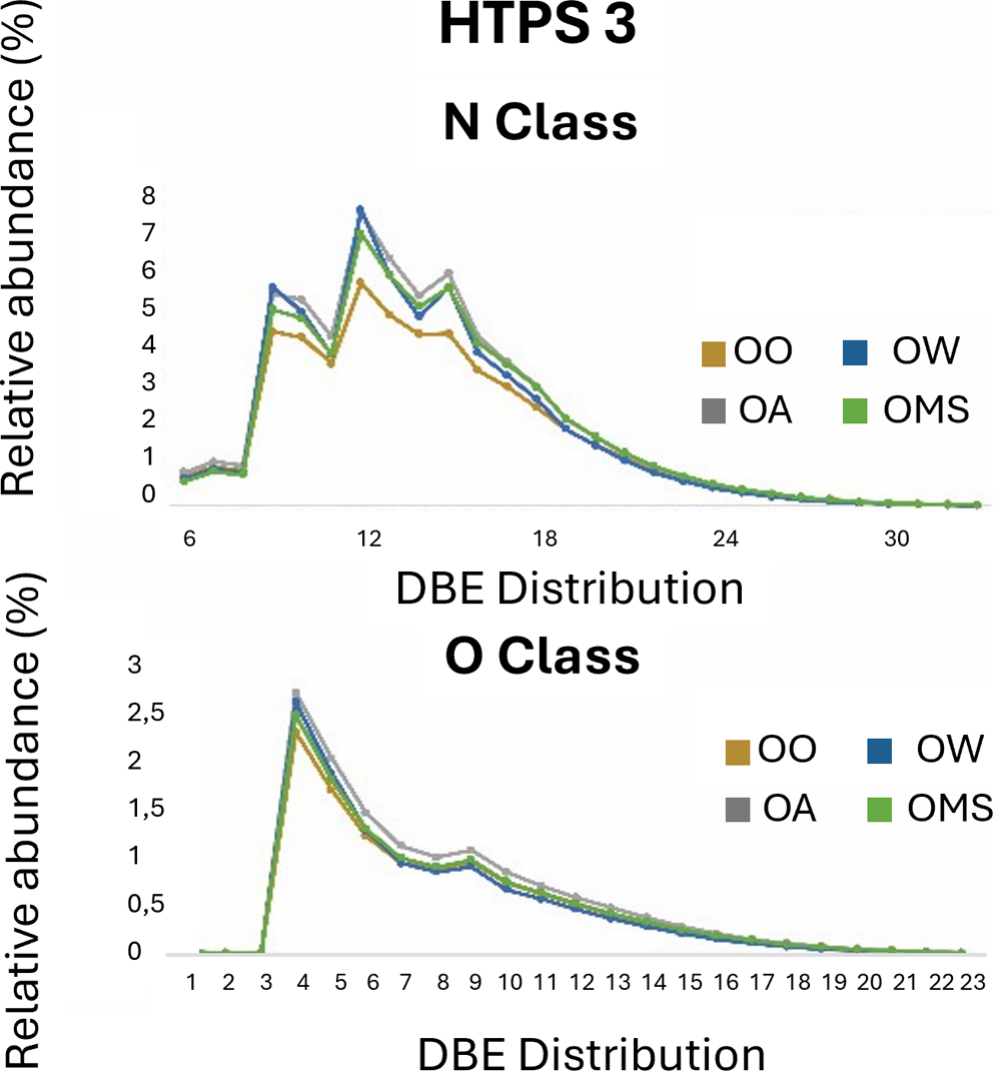
HTPS 3 DBE distribution plots of the 1 N class and O class.

Figures 21 and 23 show a significant increase in the relative abundance of the N class in the HTPS 1 and HTPS 3 oils after the core flooding tests (OA, OW, and OMS), particularly for carbazoles (DBE 9), benzocarbazoles (DBE 12), and dibenzocarbazoles (DBE 15) when compared to the original oil.

In contrast, HTPS 2 (Figure 22) shows a decrease in the relative abundance of the N class in OMS, mainly for carbazoles (DBE 9), benzocarbazoles (DBE 12), and dibenzocarbazoles (DBE 15), when compared to the original oil and also to the other recovered oils (OW and OA), which in this test remained with relatively high abundances for this DBE distribution, similar to the behavior observed in HTPS 1 and HTPS 3.

Figures 21 and 23 (HTPS 1 and HTPS 3) show the DBE distribution of the O class species ranging from 4 to 18. An increase in the relative abundance of low-DBE compounds in the oils after the core flooding tests (OA, OW, and OMS) is also observed compared with the original oil (OO), particularly for DBE values of 4, 5, and 6. This trend is more pronounced in HTPS-1.

Figure 22(HTPS 2) shows the DBE distribution of O-class species also ranging from 4 to 18; however, the increase in the relative abundance of low-DBE compounds (4, 5, and 6) occurred primarily for OA compared to OO. For the recovered OMS oil, a decrease in this DBE range was observed, while the OW profile remained practically unchanged, relative to the original oil.

### Effects of Modified Cationic Starch Characteristics on Polar Adsorption

The oils studied exhibited changes in their polar composition, mainly in relation to the nitrogen compounds. In cases where a reduction in nitrogen compounds was observed in the recovered oils (OA, OW, and OMS), this suggests that these compounds may have been preferentially adsorbed onto the rock minerals (geochromatography) or removed by the injected solutions through the water washing process, in which their solubility in water predominates.

Furthermore, the observed changes in the composition of polar compounds after flooding with modified cationic starches indicate that such polymers affect the recovery of basic and acidic polar compounds from the oil in a specific manner since the oils recovered with the modified cationic starches were the most affected across all tests. These results suggest that the wettability alteration mechanism is associated with the preferential removal of polar compounds, possibly mediated by ionic pair formation,^5^ and may explain the behavior observed for HTPS 2, whose recovered oil showed a marked removal of nitrogen compounds.

A well-established consensus in the literature highlights the role of polymer average molar mass in controlling adsorption behavior.^59,61,62^ In order to perform a more in-depth analysis of the influence of the modified starches on the removal of basic and acidic nitrogen compounds from the oils and possibly on their adsorption onto carbonate rock, the relationship between the relative abundances of the N class in the oils recovered with modified cationic starches (HTPS 1, HTPS 2, and HTPS 3) and the average molar mass of the polymers (Figure 24), as well as their degree of substitution (DS; Figure 25), was investigated.

**Figure 24:**
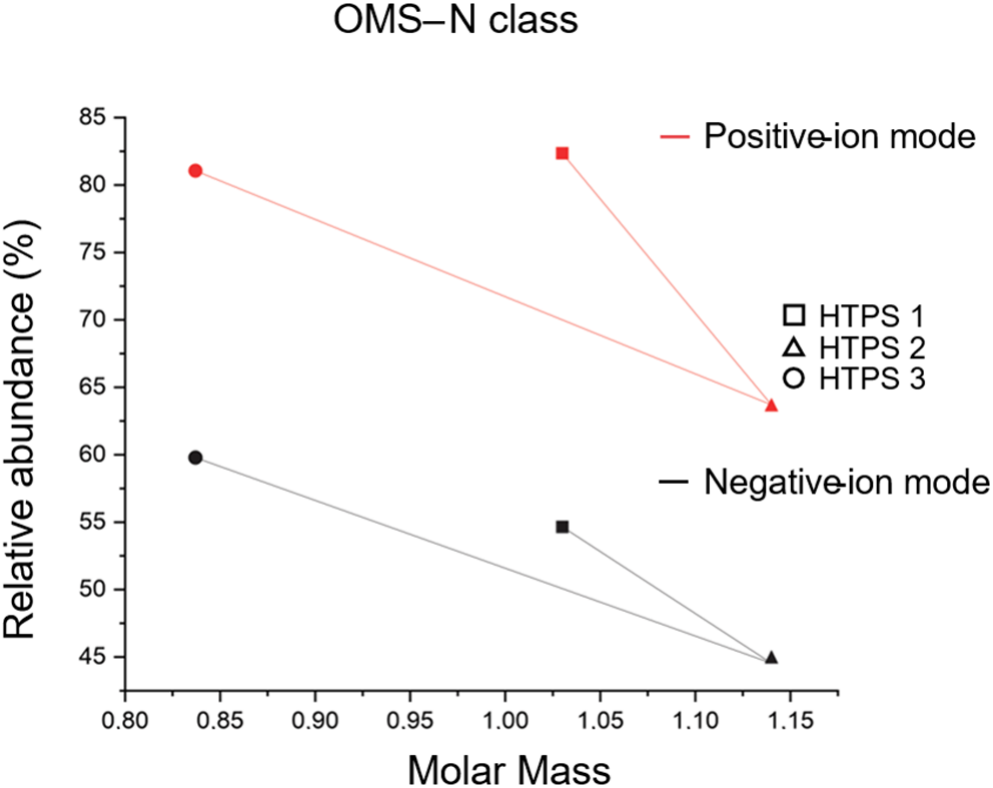
Effects of the MM of the polymers on basicity and removal of acid nitrogen in the analyzed oils (HTPS 1, HTPS 2, and HTPS 3).

**Figure 25:**
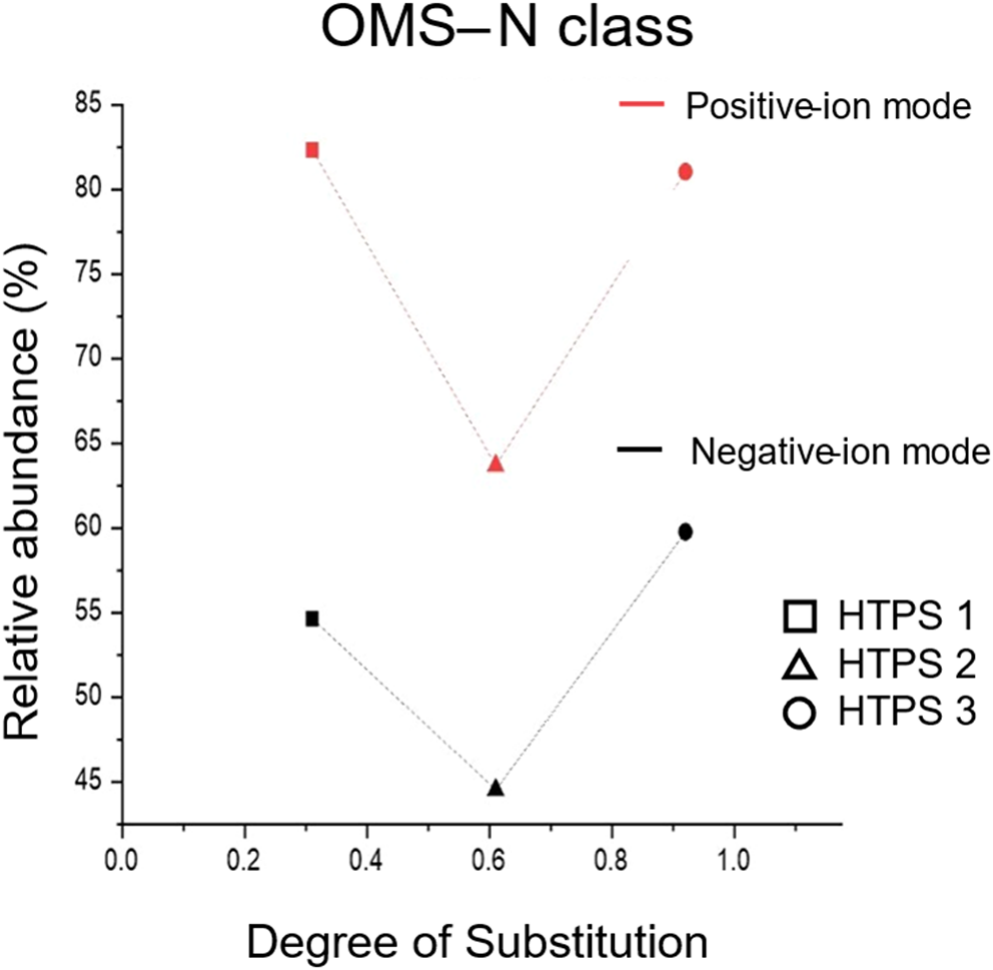
Effects of the polymer degree of substitution on basicity and removal of acid nitrogen in the analyzed oils (HTPS 1, HTPS 2, and HTPS 3).

What can be observed from Figure 24 is a clear inverse relationship among the relative abundance of nitrogen species in the three oils and the average molar mass of the polymers. Specifically, the higher the average molar mass of the starch used (HTPS 2 > HTPS 1 > HTPS 3), the lower the relative abundance of N species in the recovered oil (HTPS 2 < HTPS 1 < HTPS 3), in both positive- and negative-ion modes, with a more pronounced reduction observed in the negative-ion mode.

Conversely, as shown in Figure 25, there is no direct relationship between the relative abundance of nitrogen species in the three oils and the degree of substitution of starches. For instance, HTPS 2 exhibits the lowest relative abundance of the N class despite having an intermediate degree of substitution (degree of substitution: HTPS 1 < HTPS 2 < HTPS 3; N class relative abundance: HTPS 2 < HTPS 3 < HTPS 1), in both positive- and negative-ion modes, with the negative-ion mode consistently showing the lowest relative abundances of these species in the three oils.

The data presented here suggest that both the average molar mass and the degree of substitution of the modified starches influence the removal of polar compounds from the oil and their potential adsorption to carbonate rocks. However, although the removal of these adsorbed polar species has also been associated with the formation of ionic pairs,^59^ the results shown in Figure 25 indicate that the degree of substitution is not a determining factor for the adsorption of nitrogen compounds (either basic or acidic).

On the other hand, the molecular weight of the starches used appears to exert a predominant influence on the removal of polar nitrogen compounds (both basic and acidic) from the oil and their possible adsorption onto carbonate rocks, particularly for acidic nitrogen species (Figure 24). Notably, the oil recovered with modified cationic starch in HTPS 2 exhibited the best charge-to-mass balance among the systems evaluated in this study.

These findings are consistent with the literature,^59,63^ which indicates that acidic species are key parameters controlling wettability in carbonate reservoirs, since carbonate rocks tend to preferentially adsorb acidic compounds over basic ones. This behavior occurs because carbonate minerals are typically positively charged under alkaline pH conditions due to carbonate dissolution in formation water.

Additionally, the present results agree with previous findings,^21^ demonstrating that HTPS 2 exhibited the greatest ability to alter the wettability of carbonate rocks while also achieving the highest enhanced oil recovery factor among the three experiments.

## Conclusions

The EOR displacement results demonstrated that injecting one pore volume of a 1 wt % aqueous solution of each HTPS derivative promoted additional oil displacement. Among them, HTPS 2 achieved the highest oil recovery factor and exhibited the largest pressure differential, highlighting the importance of viscosity enhancement in improving sweeping efficiency and altering rock wettability. For future developments, it is suggested that the most promising HTPS derivatives for EOR applications should possess both a high cationic degree of substitution and high molecular weight, as observed for HTPS 2.

The distribution of basic and acidic polar compounds in the oil was affected by both waterflooding and modified cationic starch injection (EOR). In general, all tests indicated a more pronounced impact on nitrogen-containing compounds and, to a lesser extent, on oxygenated species, particularly in analyses conducted in negative-ion mode. Overall, when ranking the performance of the modified cationic starches, HTPS 2 provided the most favorable outcomes regarding polar compound distribution in the recovered oils, followed by HTPS 3 and HTPS 1.

HTPS 2 showed the lowest relative abundances of nitrogen species in the recovered oils (OW and OMS) in both positive- and negative-ion modes. In this test, nitrogen compounds and their analogues were likely removed by the injected water or, more significantly, adsorbed onto the carbonate rock surface, given the observed depletion of basic nitrogen compounds with higher aromaticity, particularly nitrogen acids such as carbazoles (DBE 9), benzocarbazoles (DBE 12), and dibenzocarbazoles (DBE 15) in the oil after the EOR. The results also suggest that starch molecular weight had a marked influence on the compositional variations of nitrogen compounds in the recovered oils and consequently on their adsorption behavior in the carbonate matrix, while the degree of substitution contributed to a lesser extent. Among the tested samples, HTPS 2 oil presented the most balanced charge-to-mass distribution, especially in the negative-ion mode, indicating that the starch molecular weight strongly influences the removal and adsorption of acidic nitrogen species.

Regarding oxygenated compounds (detected in the negative-ion mode), the oils recovered from HTPS 2 and HTPS 3, particularly the OMS from HTPS 3, showed a lower tendency to form stable emulsions and therefore a reduced impact on flow assurance, as evidenced by their lower O/O_2_ ratios compared to HTPS 1. Analysis of the O_2_ class (DBE 1) also revealed similar reductions in relative abundance for HTPS 2 and HTPS 3 oils, in contrast to HTPS 1, which exhibited markedly higher values, especially for the OMS sample.

The study of compositional changes in petroleum following secondary (waterflooding) and tertiary (EOR) recovery processes, particularly through the analysis of polar compounds, demonstrates that detailed chemical characterization of recovered oils is essential to properly assess their quality. These recovery processes can significantly modify the oil composition at the molecular level. Among them, modified cationic starch injection (EOR) proved to be one of the most effective methods for altering the petroleum chemical composition. The molecular weight of the starches played a major role in these transformations, particularly when high-molecular-weight starches were combined with carbonate rocks, which have a higher capacity to retain polar acidic species.

Further investigation is required to better understand the mechanisms controlling polar compound adsorption during modified cationic starch injection, particularly regarding starch structural characteristics and rock–fluid interactions.

## ∥

Federal Institute of Education, Science and Technology of Rio de Janeiro (Instituto Federal do Rio de Janeiro – IFRJ), Nilópolis, RJ 26530–060, Brazil

## Abbreviations

API: American Petroleum Institute
CAT: cationic starch test
DBE: double-bond equivalent
DS: degree of substitution
ESI: electrospray ionization
EOR: enhanced oil recovery
FT-ICR MS: Fourier transform ion cyclotron resonance mass spectrometry
MW: molecular mass
OMS: oil recovered with modified cationic starch
OO: original oil
OA: oil after aging
OW: oil recovered with brine
TAN: total acid number
